# CSF1R inhibitor JNJ-40346527 attenuates microglial proliferation and neurodegeneration in P301S mice

**DOI:** 10.1093/brain/awz241

**Published:** 2019-08-26

**Authors:** Renzo Mancuso, Gemma Fryatt, Madeleine Cleal, Juliane Obst, Elena Pipi, Jimena Monzón-Sandoval, Elena Ribe, Laura Winchester, Caleb Webber, Alejo Nevado, Tom Jacobs, Nigel Austin, Clara Theunis, Karolien Grauwen, Eva Daniela Ruiz, Amrit Mudher, Marta Vicente-Rodriguez, Christine A Parker, Camilla Simmons, Diana Cash, Jill Richardson, NIMA Consortium, Edward T Bullmore, Junaid Bhatti, Samuel J Chamberlain, Marta M Correia, Anna L Crofts, Amber Dickinson, Andrew C Foster, Manfred G Kitzbichler, Clare Knight, Mary-Ellen Lynall, Christina Maurice, Ciara O'Donnell, Linda J Pointon, Peter St George Hyslop, Lorinda Turner, Petra Vertes, Barry Widmer, Guy B Williams, B Paul Morgan, Claire A Leckey, Angharad R Morgan, Caroline O'Hagan, Samuel Touchard, Jonathan Cavanagh, Catherine Deith, Scott Farmer, John McClean, Alison McColl, Andrew McPherson, Paul Scouller, Murray Sutherland, H W G M (Erik) Boddeke, Jill C Richardson, Shahid Khan, Phil Murphy, Christine A Parker, Jai Patel, Declan Jones, Peter de Boer, John Kemp, Wayne C Drevets, Jeffrey S Nye, Gayle Wittenberg, John Isaac, Anindya Bhattacharya, Nick Carruthers, Hartmuth Kolb, Carmine M Pariante, Federico Turkheimer, Gareth J Barker, Heidi Byrom, Diana Cash, Annamaria Cattaneo, Antony Gee, Caitlin Hastings, Nicole Mariani, Anna McLaughlin, Valeria Mondelli, Maria Nettis, Naghmeh Nikkheslat, Karen Randall, Hannah Sheridan, Camilla Simmons, Nisha Singh, Victoria Van Loo, Marta Vicente-Rodriguez, Tobias C Wood, Courtney Worrell, Zuzanna Zajkowska, Niels Plath, Jan Egebjerg, Hans Eriksson, Francois Gastambide, Karen Husted Adams, Ross Jeggo, Christian Thomsen, Jan Torleif Pederson, Brian Campbell, Thomas Möller, Bob Nelson, Stevin Zorn, Jason O'Connor, Mary Jane Attenburrow, Alison Baird, Jithen Benjamin, Stuart Clare, Philip Cowen, I-Shu (Dante) Huang, Samuel Hurley, Helen Jones, Simon Lovestone, Francisca Mada, Alejo Nevado-Holgado, Akintayo Oladejo, Elena Ribe, Katy Smith, Anviti Vyas, Zoe Hughes, Rita Balice-Gordon, James Duerr, Justin R Piro, Jonathan Sporn, V Hugh Perry (PI, Madeleine Cleal, Gemma Fryatt, Diego Gomez-Nicola, Renzo Mancuso, Richard Reynolds, Neil A Harrison, Mara Cercignani, Charlotte L Clarke, Elizabeth Hoskins, Charmaine Kohn, Rosemary Murray, Lauren Wilcock, Dominika Wlazly, Howard Mount, Declan N C Jones, Simon Lovestone, Diego Gómez-Nicola, V Hugh Perry

**Affiliations:** 1 Biological Sciences, University of Southampton, Southampton General Hospital, Southampton, UK; 2 Department of Physiology Anatomy and Genetics, University of Oxford, Sherrington Building, Parks Road, Oxford OX1 3PT, UK; 3 UK Dementia Research Institute, Cardiff University, Hadyn Ellis Building, Maindy Road, Cardiff, CF24 4HQ, UK; 4 Janssen Research and Development, Turnhoutseweg 30, box 270, 2340 Beerse 1, Belgium; 5 Janssen Neuroscience Research and Development, Janssen Pharmaceutical Companies of Johnson and Johnson, Turnhoutseweg 30, 2340, Beerse, Belgium; 6 Department of Neuroimaging, Institute of Psychiatry, Psychology and Neuroscience, King’s College London, London, UK; 7 Experimental Medicine Imaging, GlaxoSmithKline, Gunnels Wood Road, Stevenage, SG1 2NY, UK; 8 Neurosciences Therapeutic Area, GlaxoSmithKline R&D, Stevenage, UK; 9 Janssen Neuroscience External Innovation, Johnson and Johnson Innovation Centre, One Chapel Place, London, W1G 0BG, UK; 10 Oxford Health NHS Foundation Trust, Oxford, UK

**Keywords:** Alzheimer’s disease, microglia, neuroinflammation, tau, CSF1R

## Abstract

Neuroinflammation and microglial activation are significant processes in Alzheimer’s disease pathology. Recent genome-wide association studies have highlighted multiple immune-related genes in association with Alzheimer’s disease, and experimental data have demonstrated microglial proliferation as a significant component of the neuropathology. In this study, we tested the efficacy of the selective CSF1R inhibitor JNJ-40346527 (JNJ-527) in the P301S mouse tauopathy model. We first demonstrated the anti-proliferative effects of JNJ-527 on microglia in the ME7 prion model, and its impact on the inflammatory profile, and provided potential CNS biomarkers for clinical investigation with the compound, including pharmacokinetic/pharmacodynamics and efficacy assessment by TSPO autoradiography and CSF proteomics. Then, we showed for the first time that blockade of microglial proliferation and modification of microglial phenotype leads to an attenuation of tau-induced neurodegeneration and results in functional improvement in P301S mice. Overall, this work strongly supports the potential for inhibition of CSF1R as a target for the treatment of Alzheimer’s disease and other tau-mediated neurodegenerative diseases.

## Teaser

Microglia have been implicated in amyloid beta-induced neuropathology, but their role in tau-induced neurodegeneration remains unclear. Mancuso *et al.* report that blockade of microglial proliferation by CSF1R inhibitor JNJ-40346527 modifies brain inflammation and ameliorates disease progression in P301S tauopathy mice. CSF1R inhibition may have therapeutic potential in tau-mediated neurodegenerative diseases.

## Introduction

Alzheimer’s disease is the most common form of dementia affecting roughly 6–7% of the population aged 65, and up to 30% of people aged 85 and over (Hebert *et al.*, 2003; Qiu *et al.*, 2009). From a pathological perspective, Alzheimer’s disease is characterized by extensive accumulation of extracellular amyloid-β soluble peptides and plaques, and intracellular neurofibrillary tau tangles, together with gliosis, neuronal dystrophy and loss, and vascular alteration (Sala Frigerio and De Strooper, 2016). The presence of amyloid plaques and neurofibrillary tangles is accompanied by a robust innate immune response characterized by microglial activation (Akiyama *et al.*, 2000; Edison *et al.*, 2008) and expression of pro-inflammatory cytokines and chemokines (Dickson *et al.*, 1993; Fernández-Botrán *et al.*, 2011).

The critical contribution of inflammation to Alzheimer’s disease pathogenesis has been further implicated by recent genome-wide association studies (GWAS), which highlight multiple immune-related genes in association with Alzheimer’s disease (Efthymiou and Goate, 2017), including the colony-stimulating factor 1 receptor (*CSF1R*) (Sassi *et al.*, 2018). This evidence indicates that neuroinflammation and microglial activation are key drivers of Alzheimer’s disease pathology. Interestingly, CSF1R signalling mediates microglial proliferation and survival, and both upregulation of CSF1R and increased proliferation of microglia have been reported in post-mortem samples from patients with Alzheimer’s disease (Akiyama *et al.*, 1994; Gomez-Nicola *et al.*, 2013; Olmos-Alonso *et al.*, 2016).

The importance of neuroinflammation in Alzheimer’s disease is also strongly supported by experimental data from several rodent models (Heneka *et al.*, 2015). We showed previously that inhibition of microglial proliferation by pharmacologically blocking CSF1R ameliorates disease progression in the APP/PS1 (Olmos-Alonso *et al.*, 2016) model of Alzheimer’s disease, but also multiple experimental models of neurodegenerative disease, including prion disease (Gomez-Nicola *et al.*, 2013) and amyotrophic lateral sclerosis (ALS) (Martinez-Muriana *et al.*, 2016). Similar results have been shown using microglial depletion strategies in 3xTg-ADD (Dagher *et al.*, 2015) and 5xTg-AD models (Spangenberg *et al.*, 2016; Sosna *et al.*, 2018). Partial depletion of microglia has been also recently tested in aged Tg2510 mice, but failed to modify tau pathology (Bennett *et al.*, 2018).

Recent single-cell transcriptomic analysis from Alzheimer’s disease mice brains has shed some light on the transcriptional signature of plaque-associated microglia (named ‘disease-associated microglia’, DAM), in which there is a two-step switch from a homeostatic (*Tmem119*, *P2ry12*, *Cx3cr1* signature) to a pathology-associated phenotype (*Apoe*, *Trem2*, *Csf1*, *Itgax* signature), with TREM2 as a major phenotypic modulator (Keren-Shaul *et al.*, 2017). This microglial transcriptomic profile has been also observed in pure tauopathy models including P301S and P301L mice (Friedman *et al.*, 2018). However, whether microglial proliferation and associated phenotypic changes influence tau pathology, the other major proteinopathy of Alzheimer’s disease, and other tauopathies or tau-mediated neurodegeneration, is not clear.

In this study, we describe the effects of the selective clinically available CSF1R inhibitor JNJ-40346527 (JNJ-527) on microglia in chronic neurodegeneration. JNJ-527 is a potent inhibitor of CSF1R tyrosine kinase activity with high selectivity over similar related kinases, such as KIT and FLT3 (CSF1R IC_50_ = 3.2 nM; KIT IC_50_ = 20 nM; FLT3 IC_50_ = 190 nM) (Genovese *et al.*, 2015; Von Tresckow *et al.*, 2015). Overall, we: (i) demonstrate the anti-proliferative effects of JNJ-527 upon microglia in the ME7 prion model and provided potential CNS biomarkers for the clinical investigation of the compound, including efficacy assessment by TSPO autoradiography and CSF proteomics; and (ii) show for the first time that blockade of microglial proliferation and modification of microglial phenotype may lead to the amelioration of tau pathology and attenuation of neurodegeneration. This work strongly supports the potential of inhibition of CSF1R as a target for the treatment of Alzheimer’s disease and other tau-mediated neurodegenerative diseases.

## Materials and methods

### *In vitro* assessment of CSF1R phosphorylation

The N13 murine microglia cell line (Righi *et al.*, 1991) was cultured in Dulbecco’s modified Eagle’s medium (DMEM; Thermo Fisher Scientific), supplemented with 10% foetal bovine serum and 50 U/ml penicillin/streptomycin (Thermo Fisher Scientific). Cells were maintained in T75 flasks at 37°C in a 5% CO_2_ humidified atmosphere. Cells were plated at a density of 2 × 10^5^ cells/cm^2^ in 6-well plates and cultured overnight to allow adherence. Cells were kept in serum-free medium for 4 h prior to stimulation and then incubated without or with 0.1, 1, 10, 100 or 1000 nM of JNJ-527 for 30 min. Recombinant CSF1 (100 ng/ml, R&D Systems) was added to respective wells for 5 min, after which cells were immediately lysed in RIPA buffer (Thermo Fisher Scientific), supplemented with protease and phosphatase inhibitor cocktails (Roche, Thermo Fisher Scientific). Protein lysates were concentrated using Microcon-10 kDa Centrifugal Filter Units (Merck Millipore), according to manufacturer’s instructions and protein concentration was determined using the Pierce BCA Protein Assay Kit (Thermo Fisher Scientific). For estimation of IC50, values for CSF1R and ERK1/2 phosphorylation were modelled in a non-linear regression curve using GraphPad prism.

### Experimental model of prion disease

C57BL/6 J (Harlan) mice were bred and maintained in local facilities. Mice were housed in groups of 4–10, under a 12-h light/12-h dark cycle at 21°C, with food and water *ad libitum.* To induce prion disease, 8–10-week-old mice were anaesthetized with a ketamine/xylazine mixture (85 and 13 mg/kg), and 1 μl of either ME7-derived (ME7 animals) brain homogenate (10% w/v) or normal brain homogenate (NBH animals) was injected stereotaxically and bilaterally at the coordinates from bregma: anteroposterior, −2.0 mm; lateral, ±1.7 mm; depth, −1.6 mm (Gomez-Nicola *et al.*, 2013). All animals were at 12 weeks post-induction at the beginning of the treatment period. When required, mice received intraperitoneal BrdU (7.5 mg/ml, 0.1 ml/10 g weight in sterile saline; Sigma-Aldrich) for the 2 days before the end of the experiment. All animal studies were ethically reviewed and carried out in accordance with Animals (Scientific Procedures) Act 1986 and the GSK Policy on the Care, Welfare and Treatment of Animals.

### Experimental model of tauopathy

P301S transgenic mice were developed by Prof. Michel Goedert, Division of Neurobiology, University of Cambridge (Cambridge, UK). The detailed description of the animal model can be found in Allen *et al.* (2002). Homozygous P301S and non-transgenic C57BL/6 wild-type mice were used in this study. Mice were killed at 6, 12, 16 and 20 weeks of age.

### Pharmacological treatments

For short-term treatments, JNJ-527 was dissolved in 0.9% Methocel^™^ and administered daily (morning) for five consecutive days by oral gavage at doses of 3, 10, 30 and 100 mg/kg. For long term treatments (4–8 weeks), JNJ-527 was incorporated into mouse chow as previously described by Olmos-Alonso *et al.* (2016), for a final dose of 30 mg/kg with an average daily ingestion of 5 g of food per mouse. Diet composition was identical in terms of fat, protein, etc. content, with the only addition of the compound. Mouse weight and food consumption were monitored in all experiments, and no differences were found between treated and untreated groups. All the experimental groups were randomized to avoid gender and cage effects, and all the experiments were performed by blinded researchers.

### Behavioural tests

Multiple tests were performed to detect the onset and progression of behavioural abnormalities in ME7 (Boche *et al.*, 2006; Gomez-Nicola *et al.*, 2013) and P301S mice (Scattoni *et al.*, 2010): open-field locomotor activity, and motor performance on inverted screen, horizontal bar and rotarod test.

#### Locomotor activity

The open-field test was performed using activity monitor software (Med Associated). Mice were placed in individual cages of 27 × 27 cm for a period of 5 min and the total distance travelled was analysed. The average speed was used as an internal control of the mouse motor abilities.

#### Horizontal bar

The test was performed on a 38-cm long metal bar with a diameter of 0.2 cm that was supported by wooden struts to a height of 49 cm over a padded bench surface. Mice were held by the tail and allowed to grip the centre of the bar with their front paws only. The tail was rapidly released and time taken to fall off (60 s) or to reach one of the wooden supports (contact with forepaw) was recorded.

#### Inverted screen

The inverted screen is a 43-cm square of wire mesh consisting of 12-mm squares of 1-mm diameter wire. It is surrounded by a 4-cm deep wooden beading, which prevents mice from climbing on to the other side (Guenther *et al.*, 2001). Mice were placed in the centre of the grid and the latency to fall was recorded. One hundred and twenty seconds was considered an arbitrary maximum.

#### Rotarod test

Motor coordination, balance and strength of the animals were assessed using the rotarod test (Brooks and Dunnett, 2009; Mancuso *et al.*, 2012). All mice were trained three times a week on the rod rotating with an acceleration of 4–40 rpm in 5 min, for a maximum of 300 s to reach the baseline level of performance (Scattoni *et al.*, 2010). Animals were then tested every 2 weeks at the same speed, and the latency to fall from the rotating rod was measured. Three hundred seconds was considered an arbitrary maximum time of remaining on the rotating rod.

### Histology

Mice were terminally anaesthetized with an overdose of sodium pentobarbital and transcardially perfused with 0.9% saline. Samples were cut in transverse serial sections (35-μm thick) with a vibrating microtome (brain samples) or cryostat (spinal cord samples, Leica). For each segment, series of six or 10 sections were sequentially collected free-floating and kept in Olmos solution at −20°C for brain and spinal cord, respectively.

To analyse motor neurons preservation, spinal cord sections were rehydrated for 1 min and stained for 2 h with an acidified solution of 3.1 mM Cresyl violet. Sections were then washed in distilled H_2_O for 1 min, dehydrated and mounted with DPX (Mancuso *et al.*, 2011, 2012). Motor neurons were identified by their localization in the ventral horn of the stained spinal cord sections and quantified following size and morphological criteria: only motor neurons with diameters larger than 20 μm and with polygonal shape and prominent nucleoli were quantified. Motor neurons present in the lateral site of both ventral horns were quantified in four serial sections of L4–L5 segments (Mancuso *et al.*, 2012, 2016; Martinez-Muriana *et al.*, 2016).

Immunohistochemistry was performed as described previously (Mancuso *et al.*, 2016; Martinez-Muriana *et al.*, 2016; Clayton *et al.*, 2017). Briefly, sections were treated with 10% methanol and 0.3% H_2_O_2_ to block for endogenous peroxidase activity (only for bright field immunohistochemistry), and with 5% normal serum + 0.5% BSA in phosphate-buffered saline (PBS) for non-specific binding. After rinses with PBS-0.1% Tween 20 (PBST), sections were incubated overnight at 4°C with rabbit anti-Iba1 (Wako, 019–19741), anti-PU.1 (Cell Signaling Technology #2266), anti-AT8 (Thermo Fisher, MN1020), anti-AT100 (Thermo Fisher, MN1060), anti-phospho-JNK (Cell Signaling Technology, #9251), anti-phospho-p38 (Cell Signaling Technology, #4511), anti-BrdU (Bio-Rad, MCA2060). After washes with PBST, sections were incubated with the appropriated biotinylated (Vector Labs) or Alexa 488- and 594-conjugated secondary antibodies (Invitrogen). For bright field immunohistochemistry, following rinses sections were incubated with Vectastain ABC complex (Vectors Labs) and visualized with diaminobenzidine (DAB) precipitation. Sections for bright field were mounted with DePeX and imaged either with a Leica DM5000B microscope coupled to a Leica DFC300FX camera, or by using an Olympus VS110 high throughput virtual microscopy system (Leica). For immunofluorescence labelling, sections were counterstained with DAPI and mounted with Mowiol/DABCO (Sigma-Aldrich) mixture. Sections were visualized on a Leica TCS-SP8 confocal system, coupled to a Leica CTR6500 camera.

### TSPO autoradiography

Mice were terminally anaesthetized with an overdose of sodium pentobarbital and transcardially perfused with 0.9% saline. Brains were harvested, frozen in isopentane at a temperature of −40°C and stored at −80°C. NBH (*n = *7), ME7 (*n = *8) and ME7 + JNJ-527 (*n = *8) mouse brains were coronally cryosectioned at 20 μm and directly mounted onto glass slides. Slides were incubated at room temperature for 30 min in 100 mM Tris-HCl containing 1 nM [3H]PK11195 (specific activity 82.7 Ci per mmol; Perkin Elmer), washed twice for 6 min in 100 mM Tris-HCl, rinsed dipping into dH_2_O and air dried. Non-specific binding was carried out in the presence of 20 µM PK11195 (Sigma-Aldrich) and 1 nM ^3^H-PK11195. The slides were exposed to tritium-sensitive film (Amersham Hyperfilm MP, GE Healthcare) in autoradiography cassettes together with a set of tritium standards ([3H]Microscale, American Radiolabeled Chemicals) for 6 weeks. Sections for specific and non-specific binding were processed together in a paired protocol. Films were developed using ECOMAX X-ray film processor (PROTEC). Quantitative analysis was performed using a MCID image analyser (Image Research), and brain structures were identified using the mouse brain atlas of Franklin and Paxinos (1997). All regions of interest (hippocampus, cortex and thalamus) were analysed by freehand drawing tools in three consecutive sections per brain.

### Protein analysis

Mice were terminally anaesthetized with an overdose of sodium pentobarbital and transcardially perfused with 0.9% saline. Spinal cord samples were homogenized in RIPA buffer (Thermo Fisher) with protease inhibitors (EASYpack, Roche) and phosphatase inhibitors (PhosSTOP, Roche). Homogenates were centrifuged at 1000*g* and the supernatant was collected. Protein was quantified using BCA assay (Thermo Fisher) following the manufacturer’s instructions.

#### Mesoscale multiplex plate-based assay

Assessment of inflammatory cytokines was performed using the V-PLEX Plus Proinflammatory Panel 1 Kit (MesoScale Discovery), following the manufacturer’s instructions.

#### Western blot

Twenty micrograms of protein of each sample were run on SDS-polyacrylamide gels (TGX gels, Bio-Rad), and transferred to nitrocellulose membranes by using the Trans-Blot® Turbo^™^ Transfer System (Bio-Rad). The membranes were blocked with 5% BSA in Tris-buffered saline (TBS) plus 0.1% Tween-20 for 1 h, and then incubated with primary antibodies at 4°C overnight. The primary antibodies used were: anti-AT8 (Thermo Fisher, MN1020), anti-AT100 (Thermo Fisher, MN1060), anti-AT180 (Thermo Fisher, MN1040) and total Tau (C-terminal, Dako A0024). After washes with PBST, sections were incubated with Alexa 488- and 594-conjugated secondary antibodies (Invitrogen). The membranes were imaged using the ChemiDoc^™^ system (Bio-Rad), and quantified with FIJI software.

#### Analysis of globular tau oligomers and neurofibrillary tangles

For the analysis of globular tau oligomers, protein extraction was performed as described previously (Cowan *et al.*, 2015). Briefly, P301S mice spinal cord (T12–L2) were homogenized in 100 μl buffer (50 mM Tris-Cl pH 7.4, 150 mM NaCl, 1% Triton Buffer). Samples were then centrifuged for 2 min at 1000*g.* The supernatant was centrifuged at 186 000*g* for 2 h at 4°C. The resulting supernatant (S1) represents the aqueous soluble fraction. The pellet was subsequently resuspended in 5% SDS/TBS buffer (50 mM Tris-HCl pH 7.4, 175 mM NaCl, 5% SDS) at room temperature and centrifuged at 186 000*g* for 2 h at 25°C. The resulting supernatant (S2) represents the water insoluble/SDS soluble fraction. The pellet from S2 was then washed by resuspension again in 5% SDS/TBS buffer (50 mM Tris-HCl pH 7.4, 175 mM NaCl, 5% SDS) and centrifuged at 186 000 *g* for 2 h at 25°C. To obtain S3, the final pellet was resuspended in 8 M urea, 8% SDS buffer (50 mM Tris-HCl, pH 7.4, 175 mM NaCl, 8% SDS, 8 M urea) and shaken for 12–18 h at room temperature. All samples were diluted in 2× Laemmli buffer and boiled for 5 min, and run on SDS-polyacrylamide gels (TGX^™^ gels, Bio-Rad) as described above. Membranes were stained using tau antibody (C-terminal, Dako A0024), and the proportion of globular tau oligomers was then calculated as the fraction of S3 in the total amount of tau (S1 + S2 + S3). For assessing for the presence of neurofibrillary tangles, P301S mice spinal cords were homogenized in 100 μl buffer (50 mM Tris-Cl pH 7.4, 150 mM NaCl, 1% Triton buffer). Samples were centrifuged for 2 min at 1000*g.* The supernatant was then centrifuged at 100 000*g* for 30 min at 25°C. The resulting supernatant (NS1) includes aqueous and detergent soluble tau, together with globular tau oligomers. The pellet from NS1 was subsequently washed by resuspended in 5% SDS/TBS buffer (50 mM Tris-HCl pH 7.4, 175 mM NaCl, 5% SDS) and centrifuged at 100 000*g* for 30 min at 25°C. The resulting supernatant (NS2) represents the insoluble neurofibrillary tangles enriched fraction. All samples were diluted in 2× Laemmli buffer and boiled for 5 min, and run on an SDS-polyacrylamide gel (TGX^™^ gels, Bio-Rad) as described above. Membranes were stained using tau antibody (C-terminal, Dako A0024), and the proportion of neurofibrillary tangles was calculated as the fraction of NS2 in the totality of tau (NS1 + NS2).

#### Meso scale discovery immunoassay for tau aggregation

A piece of the spinal cord was homogenized in 150 µl buffer H (10 mM Tris/HCl, 0.8 M NaCl, 10% sucrose, 1 mM EGTA, pH 7.6, including complete protease inhibitor (Roche) and PhosStop phosphatase inhibitor (Roche). Samples were centrifuged for 30 min at 4°C at 20 000*g* to clear debris. Supernatant was collected as total homogenate. Using the BCA method, protein concentration was determined, which was used to normalize total tau and aggregated measurements. Total tau and aggregated tau content was measured by a sandwich ELISA set up in a mesoscale discovery platform. During an overnight incubation at 4°C, 96-well plates (Multi-Array® plates, Meso Scale Discovery) were coated with either 1 or 2 µg/ml of antibody, depending on the optimal conditions of the assay. After a wash step with 0.05% Tween-20 in PBS, the plates were blocked with 0.1% casein in PBS for 2 h at room temperature with agitation at 400 rpm. After another wash step, samples were diluted 1/30 and 1/100 for the aggregation assays and 1/100 and 1/300 for the total tau assays in blocking buffer and incubated overnight at 4°C. Following the incubation, plates were washed again and incubated with the sulpho-labelled detection antibody, diluted in blocking buffer, for 2 h at room temperature. Dilution of the detection antibody is dependent on the assay. Before reading the plates with the MSD SECTOR Imager 600 (Meso Scale Discovery), plates were washed again and 150 µl of 2× MSD Read Buffer T was added. Concentration of tau (ng/ml) measured by the total tau assays was calculated by a standard curve of recombinant tau. The amount of aggregated tau was represented as arbitrary units and calculated by a linear dilution of a total homogenate preparation of a transgenic mouse with proven high amount of aggregated tau. Statistical analysis was conducted in GraphPad, using an unpaired *t*-test.

#### Aptamer capture arrays

Protein was extracted from mouse CSF for 59 mice comprising NBH *n = *29 and ME7 *n = *30, with each type receiving JNJ-527 doses: vehicle control *n = *10; 10 mg/kg *n = *10; 30 mg/kg *n = *9, 10. Five microlitres of CSF were hybridized to the SomaScan Version 3.2 array containing 3999 aptamers (SomaLogic) as described in the proprietary methods. For the statistical analysis, 1363 aptamer probes were formally validated by SomaLogic for cross reaction with mouse proteins (including the ligand of CSR1R IL-34, but not CSF1). After quality control, 1202 proteins were used for statistical analysis. Linear models were used to compare the experimental variables and discern effect of inhibitor dosage and mouse model of protein expression, whereby expression was defined as the outcome measure with model (ME7/NHB) and drug dosage (0, 10, 30) as dependent variables. Proteins with differential expression changes mirroring dosage increases were selected.

### Pharmacokinetics: sample preparation and bioanalytical method

Aliquots (10 µl) of plasma and brain homogenate (diluted 1:5 in phosphate buffer) were analysed for JNJ-527 concentrations using a method based on protein precipitation and HPLC-MS/MS analysis. To each sample, an internal standard (20 µl) and acetonitrile (150 µl) were added. Samples were mixed thoroughly (mechanical shaking for 10 min), and then centrifuged (5000*g* for 10 min at 4°C). An aliquot of supernatant (20 µl) was dispensed into a LCMS plate and 200 µl of 0.1% formic acid in methanol/water (50:50) were added. Analysis for JNJ-527 concentrations was performed using HPLC-MS/MS employing positive-ion electrospray ionization (Sciex API 4000) and a Zorbaz Eclipse Phenyl Hexyl, 3.5 μm (50 × 2.1 mm internal diameter) column. Elution was achieved at a flow rate of 0.5 ml/min with isocratic elution of 0.1% formic acid in methanol/water (85:15). The lower limit of quantification was 5–10 ng/ml for plasma and 10 ng/g for brain. The assay was linear up to 4000 ng/ml for plasma and 4000 ng/g for brain.

### Fluorescence-activated cell sorting analysis

Mice were terminally anaesthetized with an overdose of sodium pentobarbital and transcardially perfused with heparinized PBS. Spinal cord lumbar segments were harvested in PBS 2% foetal calf serum 2 mM EDTA [fluorescence-activated cell sorting (FACS) buffer], mechanically triturated and enzymatically dissociated using the Neural Tissue Dissociation Kit (P) (Mylteni). Then, samples were passed through a cell strainer of 70 μm mesh (BD2 Falcon) with FACS buffer, and centrifuged twice at 500*g* for 10 min at 4°C. After the second wash, cells were resuspended in 37% Percoll® (GE Healthcare) and centrifuged at 500*g* for 30 min at 18°C (Pino and Cardona, 2011; Grabert *et al.*, 2016). The supernatant and myelin layers were discarded, and the cell pellet enriched with microglia was resuspended in FACS buffer. Samples were split in several tubes and immunostained. Primary antibody labelling was performed for 1 h at 4°C, using the following primary antibodies: anti-CD11b (BD Bioscience) and anti-CD45 (BD Bioscience), anti-Ly6C (BD Bioscience) and anti-CD3 (BD Bioscience), adding 7-aminoactinomycin D (7-AAD) as a cell viability marker. Moreover, unstained cells and isotype-matched control samples were used to control for autofluorescence and/or non-specific binding of antibodies. Samples were run on a BD FACS Aria Flow cytometer. Data were analysed using FlowJo software.

### Analysis of gene expression by quantitative PCR

Mouse T10–L1 spinal cords were dissected from P301S and wild-type mice under a microscope. Samples were homogenized in TRIzol® reagent (Invitrogen), following the manufacturer instructions to isolate RNA and as described previously (Gomez-Nicola *et al.*, 2013; Clayton *et al.*, 2017). The isolated RNA was quantified (Nanodrop, Thermo Scientific) and retrotranscribed with iScript^TM^ cDNA Synthesis Kit (Bio-Rad). cDNA libraries were analysed by RT-PCR using iTaq^TM^ Universal SYBR® Green Supermix (Bio-Rad) for the following genes (Sigma-Aldrich): *Csf1* (NM_007778.4; forward (FW), agtattgccaaggaggtgtcag; reverse (RV), atctggcatgaagtctccattt), *Csf1r* (NM_001037859.2; FW, gcagtaccaccatccacttgta; RV, gtgagacactgtccttcagtgc), *Il34* (NM_00113 5100.1; FW, ctttgggaaacgagaatttggaga; RV, gcaatcctgtagttgatggggaag), *Spi1* (Pu.1) (NM_011355.1; FW, cagaagggcaaccgcaagaa; RV, gccgctgaactggtaggtga), *Cebpa* (NM_ 007678.3; FW, agcttacaacaggccaggtttc; RV, cggctggcgacatacagtac), *Il1b* (NM_00836 1.3; FW, cagacccaccctgca; RV, accgtttttccatcttcttct), *Tnfa* (NM_013 693; FW: aggcactcccccaaaagatg, RV: ttgctacgacgtgggctac), *Il6* (NM_031168.1; FW, tccagaaaccgctatgaagttc; RV, caccagcatcagtcccaaga) and *Gapdh* (NM_008084.2; FW, tgaacgggaagctcactgg, RV, tccaccaccctgttgctgta). Data were analysed using the ΔΔCt method, with *Gapdh* as a housekeeping gene.

### RNA sequencing

#### Library preparation and sequencing

RNA samples with RIN scores from 7.6 to 9 were reverse transcribed to cDNA. Polyadenylated tail selection was done using Total Dual RNA-Seq PolyA. cDNA was sequenced (HiSeq3000/4000) at the Wellcome Trust facility obtaining 75 bp per read. On average, 33 M reads were obtained per samples and a mapping rate of ∼85%.

#### Data processing

Transcript abundances were quantified using *Kallisto* (kallisto_linux-v0.43.1) (Bray *et al.*, 2016) and the corresponding index from the mouse reference transcriptome. The reference transcriptome consisted on both cDNA and ncDNA sequences from ensemble release 90 (Zerbino *et al.*, 2018). We filtered out sequences from chromosomes (or scaffold) other than chromosomes 1 to 22, X, Y and MT. For each gene, we obtained a relative transcript abundance (in transcripts per million, TPM) using the *tximport* function/package in R (Soneson *et al.*, 2016). We then filtered out all the genes with < 1 TPM across all samples and focused on protein coding genes. After filtering, 15 596 genes remained; this gene population was considered as our background for further analysis. In addition, we detected a similar number of genes on average in the spinal cord as among cortical samples (14 423 and 13 594, respectively).

#### Differential expression analysis

Differential expression analysis between groups (P301S versus wild-type; P301S+JNJ-527 versus P301S) were performed separately for each tissue using DESeq2 (Love *et al.*, 2014). The model included the number of genes detected per sample and RIN score (∼genes detected + RIN score + group) as covariates. The covariates showed strong correlation with the first principal components (Supplementary Fig. 1). We considered that a gene was detected when it had at least 1 TPM in a particular sample. Genes with a false discovery rate (FDR) <0.05 were considered differentially expressed.

#### Principal component analysis

Principal component analysis was based on the transformed gene expression data [Log_2_ (TPM + 1)] using the *prcomp* function from the stats package in R (R Core team, 2017) and plotted with the *scatterplot3d* function/R package (Ligges and Martin, 2003).

#### Hierarchical clustering

Similarity between gene expression profiles given as the Spearman correlation coefficients (ρ). Ward hierarchical clustering of the samples was calculated based on the Euclidean distance of 1 − ρ.

#### Gene ontology enrichment analysis

Gene ontology analysis was performed using the *RDAVIDWebService* package in R (Fresno and Fernández, 2013). All gene lists were given in Ensemble gene IDs. We adjusted our background population to our set of 15 596 genes.

#### Protein–protein interaction network

We created a combined protein–protein interaction (PPI) network from the following resources: BIOGRID Release 3.4.152 (Stark, 2006) [accessed on 11/09/2017], BIOPLEX 2.0 (Huttlin *et al.*, 2017) [15/09/2017], CORUM (Ruepp *et al.*, 2009) [14/09/2017], HITPREDICT (López *et al.*, 2015) [11/09/2017], INBIOMAP (Li *et al.*, 2016) [17/09/2017], INTACT (Hermjakob, 2004; Orchard *et al.*, 2014) [14/09/2017], MINT (Licata *et al.*, 2012) [15/09/2017], REACTOME ([18) [14/09/2017] and STRING 10.5 (Szklarczyk *et al.*, 2017) [15/09/2017]. From the STRING database, only protein links with experimental score have been included (i.e. score > 0). The combined PPI network was constructed summarizing PPIs in human Ensemble gene IDs. Therefore, to use this combined PPI network, we went from mouse Ensemble gene IDs to human Ensemble gene IDs only for the genes with a corresponding one-to-one orthologue in human [according to ensemble release 90 (Zerbino *et al.*, 2018)]. To test for more PPIs in a subset of genes, we compared 10 000 equally sized random samples of genes (with a similar degree and CDS length distribution). In each randomization, we measured the number of PPIs among the genes in the sample. An estimated *p*-value was obtained by counting the number of random samples where the number of PPI was higher than in our focus subset of genes. PPI network plot was created in Cystoscope (Shannon *et al.*, 2003).

#### Cell-type specific gene markers

We have used the cell-type specific gene markers from NeuroExpresso combined dataset (Mancarci *et al.*, 2017) (accessed on 17/12/2017) given in Gene Symbols. We also used some of the original sets of the mouse cortical subtypes identified by Tasic *et al.* (2016). The Tasic dataset comprise groups of markers for 23 GABAergic, 19 glutamatergic and seven non-neuronal types.

#### Overlap between differentially expressed genes and cell-type specific gene markers

We looked for cell-type specific gene markers among our set of differentially expressed genes. If we found at least one overlapping gene we tested if the observed overlap was higher than expected by chance. We estimated the expected overlap from 10 000 randomizations. In each randomization, a group of genes of the same size as our focus set of genes were drawn from our background population and the overlap with a specific group of gene markers was measured. A *P*-value was estimated based on the number of time that the overlap in the randomizations was higher than in our focus population. To adjust for multiple testing, we used the Benjamini and Hochberg method.

#### Differences in the average fold change

Additionally, we tested whether the average fold-change (between P301S and wild-type; or between P301S+JNJ-527 and P301S) among a specific set of gene markers was different than expected by chance. Similarly, we created 10 000 equally-sized random samples of genes and obtain their average fold-change, estimated a *P*-value based on the randomizations and adjust for multiple testing using the Benjamini and Hochberg method.

#### Cell type gene expression data

We used three independent gene expression datasets to identify cell specific gene expression. A combined microarray dataset for multiple cell types were downloaded from NeuroExpresso (Mancarci *et al.*, 2017) (accessed on 11/12/2017) and preprocessed RNA-seq gene expression data from Tasic (Tasic *et al.*, 2016) also available through NeuroExpresso. Additionally, we used the preprocessed (FPKM) RNA-seq gene expression data from the RNA-Sequencing Transcriptome and Splicing Database of Glia, Neurons, and Vascular Cells of the Cerebral Cortex (Zhang *et al.*, 2014) (accessed on 11/12/2017).

### General statistics

Data are shown as mean ± standard error of the mean (SEM) and where analysed using the GraphPad Prism 6 software package (GraphPad Software). Functional data were analysed using two-way repeated measurements ANOVA with Tukey *post hoc* test for multiple comparisons. *T*-test and one-way ANOVA was used for the histological, gene expression data, FACS analysis and protein data, when comparing two or more than two groups, respectively, followed by a Tukey *post hoc* test for multiple comparisons. Differences were considered significant at *P* < 0.05.

For the pharmacokinetic/pharmacodynamics analysis of JNJ-527, the number of Iba1-BrdU (cells/mm^2^), *y_i_*, is assumed to follow a normal distribution

(1) $$y_{i}\;∼\;N\;(\mu ,\;\sigma^{2})$$

where the mean *μ* is modelled using a hill function:

(2) $$\mu =\;E_{0}\;\left(1-\;\frac{C^{\gamma }}{C^{\gamma }+\;EC_{50}^{\gamma }}\right)$$

where *E*_0_ represents the untreated number of cells, i.e. the vehicle data. Further, a maximal attainable inhibition of 100% is assumed, which means that an extremely large concentration *C* would lead to a complete eradication of the cells. Further, EC50 corresponds to the concentration leading to a 50% decrease of the number of cells, which is often referred to as the potency, and γ corresponds to the steepness of this slope. Both the two last parameters are fitted on the log-scale to reflect its underlying statistical properties and subsequently back-transformed. The data are fitted both using the plasma and the brain concentrations as explanatory variables. The model is fitted using the procedure nlmixed in SAS. The model prediction and its 95% confidence intervals were graphically represented using R.

### Data availability

The full protein and differential expression analyses performed on CSF and brain or spinal cord tissue are available in Supplementary Tables 1 and 2, respectively. The data and code used in this work will be available upon request.

## Results

We used two independent models to address two different but complementary aspects of the preclinical testing of JNJ-527. First, we used the ME7-prion model for testing the basic pharmacology of the compound. The rapid increase in microglia number and responsiveness to CSF1R inhibitors of the prion model (Gomez-Nicola *et al.*, 2013) was the most appropriate for testing short- and long-term efficacy, defining pharmacokinetic/pharmacodynamics parameters, and providing a set of readouts that could be directly transferred for the clinical testing of the compound. We then moved to P301S mice to test the efficacy of JNJ-527 in a model of tau-induced neurodegeneration, which is particularly relevant for Alzheimer’s disease, frontotemporal dementia (FTD) and other tau pathologies. Whereas P301S mice were generated as a model of FTD, they show predominant tau accumulation in the spinal cord and brainstem, and develop robust motor neuron pathology and motor deficits (Allen *et al.*, 2002; Scattoni *et al.*, 2010). Previous reports showed strong tau phosphorylation, neurodegeneration and marked neuroinflammatory changes in the spinal cord of these mice, that we expanded further in the present study (Allen *et al.*, 2002; Bellucci *et al.*, 2004). Despite some evidence of neurodegeneration in brain areas such as perirhinal cortex (Yang *et al.*, 2015) or superficial layers of the motor cortex (Hampton *et al.*, 2010), we focused our attention on the spinal cord, as we believe it gives rise to readily assayed behavioural and cellular phenotype to dissect the contribution of microglial proliferation on tau-induced neurodegeneration.

### JNJ-527 prevents CSF1R phosphorylation in N13 microglial cells

**Figure 1:**
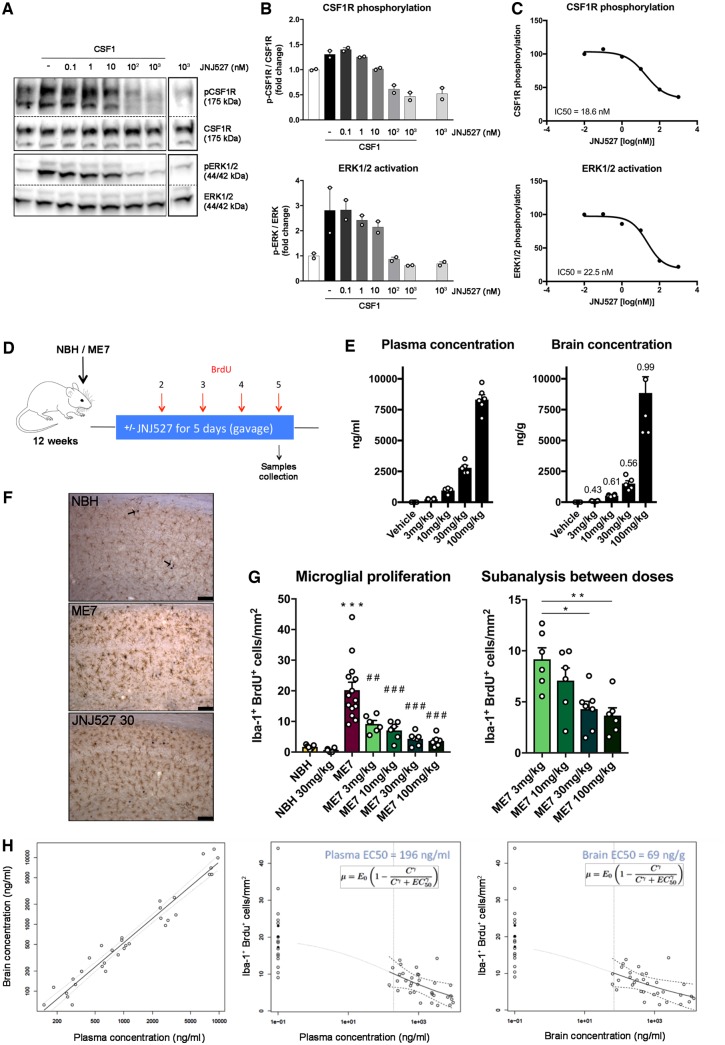
**JNJ-527 prevents CSF1R phosphorylation *in vitro* and blocks microglial proliferation *in vivo.*** (**A**) Representative western blot of N13 cells pretreated with increasing concentrations of JNJ-527 and stimulated with CSF1. (**B**) Quantification shows that JNJ-527 inhibits CSF1-R phosphorylation and downstream ERK1/2 activation at concentrations higher than 10 nM. Experiments were performed in triplicate, and values were corrected by the untreated control (white bars) and expressed as mean ± SEM. (**C**) JNJ-527 IC50 is within 18.6 and 22.5 nM. (**D**) Schematic representation of the experimental design used for assessing JNJ-527 efficacy in blocking microglial proliferation (NBH *n = *8, NBH+JNJ-527 30 mg/kg *n = *6, ME7 *n = *14, ME7+JNJ-527 3 mg/kg *n = *6, ME7+JNJ-527 10 mg/kg *n = *6, ME7+JNJ-527 30 mg/kg *n = *8, ME7+JNJ-527 100 mg/kg *n = *6). Mice were treated with JNJ-527 at different doses (3, 10, 30 and 100 mg/kg) for 5 days, with four daily injections of BrdU to quantify microglial proliferation. (**E**) Levels of the JNJ-527 were analysed at the end of the treatment. Both plasma and brain concentration linearly correlated with the treatment dose, with T/P ratios of 0.5–1 (see numbers above columns). (**F** and **G**) JNJ-527 treatment significantly inhibited microglial proliferation in ME7 mice. Images represent Iba-1 (DAB, brown) and BrdU (alkaline phosphatese, blue) immunohistochemistry with arrows pointing to Iba-1+ BrdU+ proliferating microglia. Quantification of proliferating microglia (Iba-1+ BrdU+ cells/mm^2^) showed a significant reduction at all doses tested. Subsequent analysis between different doses revealed a grester effect at 30 and 100 mg/kg. (**H**) Pharmacokinetic/pharmacodynamics (PK/PD) model generated from the microglial proliferation data showed an EC50 of 196/ml and 69 ng/g calculated from plasmatic and brain compound concentration, respectively. (*P < 0.05, **P < 0.01 and ***P < 0.001 versus NBH; ^#^*P < *0.05, ^##^*P < *0.01 and ^###^*P < *0.001 versus ME7).

First, we characterized the effect of the selective CSF1R inhibitor JNJ-527 on CSF1R activation *in vitro.* We stimulated N13 murine microglia cells with CSF1 (100 ng/ml) to induce tyrosine phosphorylation of CSF1R. Pre-incubation of the cells with a range of concentrations of JNJ-527 (0.1–10^3 ^nM) resulted in a dose-dependent decrease of CSF1R activation and a concurrent reduction of ERK1 and ERK2 phosphorylation, which are prominent intracellular signalling pathways downstream of CSF1R (Fig. 1A and B). We then generated a dose response curve for the effect of JNJ-527 on CSF1R and ERK1/2, and determined the IC50 to be 18.6 nM for inhibition of CSF1R and 22.5 nM for ERK1/2 (Fig. 1C). This demonstrates that JNJ-527 is able to prevent CSF1R phosphorylation, and activation of subsequent downstream pathways.

### JNJ-527 does not affect microglial numbers when administered below 100 mg/kg

Having demonstrated that JNJ-527 is an effective inhibitor of CSF1R *in vitro*, we turned to *in vivo* experiments and sought to determine its impact on the microglia population in the healthy brain. We performed a short-term study by daily administration of JNJ-527 to naïve mice by oral gavage for five consecutive days and at four different doses: 3, 10, 30, 100 mg/kg, and vehicle per os (p.o.). In the brain, JNJ-527 significantly diminished the number of microglia (total CD45+ CD11b+ cells) only at the highest dose tested of 100 mg/kg (Supplementary Fig. 2C). In contrast, we found that JNJ-527 depleted up to 50% of patrolling blood monocytes at every dose tested (CD45+ CD11b^high^ Ly6C^intermediate/low^ cells) with only a tendency for a reduction in the proportion of inflammatory monocytes (Ly6C^high^ cells) at 100 mg/kg (*P = *0.1) (Supplementary Fig. 2D). These data suggest that JNJ-527 administered below 100 mg/kg does not alter the dynamics of the homeostatic microglial population.

### CSF1R inhibition by JNJ-527 blocks microglial proliferation in ME7-prion mice

We then sought to assess the effect of JNJ-527 on microglial proliferation. Previously, we have demonstrated that microglial proliferation and associated phenotypic changes play a crucial role in the pathology of ME7-prion disease, and that this process is driven by the activation of CSF1R (Gomez-Nicola *et al.*, 2013). The rapid expansion of the microglial population in this model makes it a suitable platform to determine drug efficacy in short-term studies (Gomez-Nicola *et al.*, 2013). Therefore, we assessed the potential of JNJ-527 to block microglial proliferation *in vivo* in ME7-prion mice. Based on previous experiments from our group (Gomez-Nicola *et al.*, 2013), we performed a dose-response experiment by treating ME7 mice from 12 weeks post-induction (wpi) for five consecutive days with JNJ-527 at doses of 3, 10, 30, 100 mg/kg p.o., and vehicle. Mice also received four daily injections of BrdU to trace proliferating cells during the treatment period (Fig. 1D). We analysed the concentration of the compound in brain and plasma and found a linear dose dependent increase in JNJ-527 exposure, with an average brain to plasma ratio of 0.65 (Fig. 1E). We then assessed the impact of CSF1R blockade by JNJ-527 on microglial proliferation. We found that JNJ-527 significantly inhibited microglial proliferation (Iba1+ BrdU+ cells) in the hippocampus of ME7 mice from 3 mg/kg, reaching a maximum effect of 80% inhibition at 30 mg/kg (Fig. 1F and G). Based on these data, we generated a sigmoid E_max_ pharmacokinetic/pharmacodynamics model for inhibition of microglial proliferation and determined that JNJ-527 EC50 was 196 ng/ml or 69 ng/g for plasma and brain exposures, respectively (Fig. 1H). Overall, we demonstrated that JNJ-527 administered at 30 mg/kg significantly blocks microglial proliferation in ME7-prion mice, without altering the dynamics of the population in the healthy brain (Supplementary Fig. 2C and D). Therefore, we used a dose of 30 mg/kg for all subsequent experiments.

### Inhibition of CSF1R by JNJ-527 limits microglial expansion and attenuates behavioural deficits in ME7-prion mice

**Figure 2:**
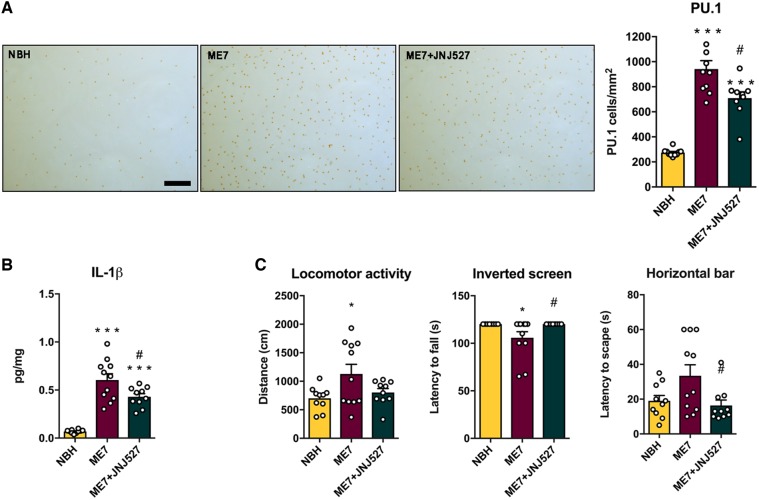
**Inhibition of CSF1R by JNJ-527 reduces microglial expansion and restores behavioural alterations in ME7 mice.** (**A**) Long-term JNJ-527 resulted in a significant reduction in the number of microglia (PU.1) in CA1 of the hippocampus. Representative images of PU.1 staining in CA1 hippocampus of NBH, ME7 and ME7+JNJ-527 mice. Scale bar = 50 μm. (**B**) JNJ-527 treatment diminished the expression of IL1-β and (**C**) attenuated the hyperactive behaviour (locomotor activity) and completely prevented the onset of motor deficits (inverted screen and horizontal bar). For all quantifications, NBH *n = *10, ME7 *n = *8, ME7+JNJ-527 *n = *9. Values are mean ± SEM. *P < 0.05 ***P < 0.001 versus NBH; ^#^*P < *0.05 versus ME7.

After determining the optimal treatment dose of JNJ-527, we aimed to confirm that the blockade of microglial proliferation translates into a long-term effect when tested under pathological conditions. We performed a 4-week treatment with JNJ-527 at 30 mg/kg in ME7-prion mice from 12 wpi. We selected this treatment regime based on our previous work showing a 4-fold increase in the number of microglia after 4 weeks of prion induction (Gomez-Nicola *et al.*, 2013). Long-term blockade of CSF1R significantly reduced the density of microglia in CA1 of the hippocampus of ME7-prion mice (PU.1+ cells) by up to 30% (Fig. 2A). This was accompanied by a significant reduction in the expression of IL1-β (Fig. 2B), but not other inflammatory cytokines (Supplementary Fig. 3). Previously, we have shown that ME7 mice develop a highly reproducible disease course characterized by an early onset associated with anhedonia (12 wpi), and followed by hyperactivity and motor dysfunction (Guenther *et al.*, 2001; Boche *et al.*, 2006; Gomez-Nicola *et al.*, 2013). The reduction in the microglial population induced by JNJ-527 led to an attenuation of hyperactivity and a complete prevention of motor deficits (Fig. 2C).

Overall, long-term blockade of CSF1R by JNJ-527 was able to limit the expansion of the microglial population, modify the inflammatory response and ameliorate the disease course *in vivo* in ME7-prion mice.

### Proof of target engagement and efficacy for the potential clinical assessment of JNJ-527

**Figure 3:**
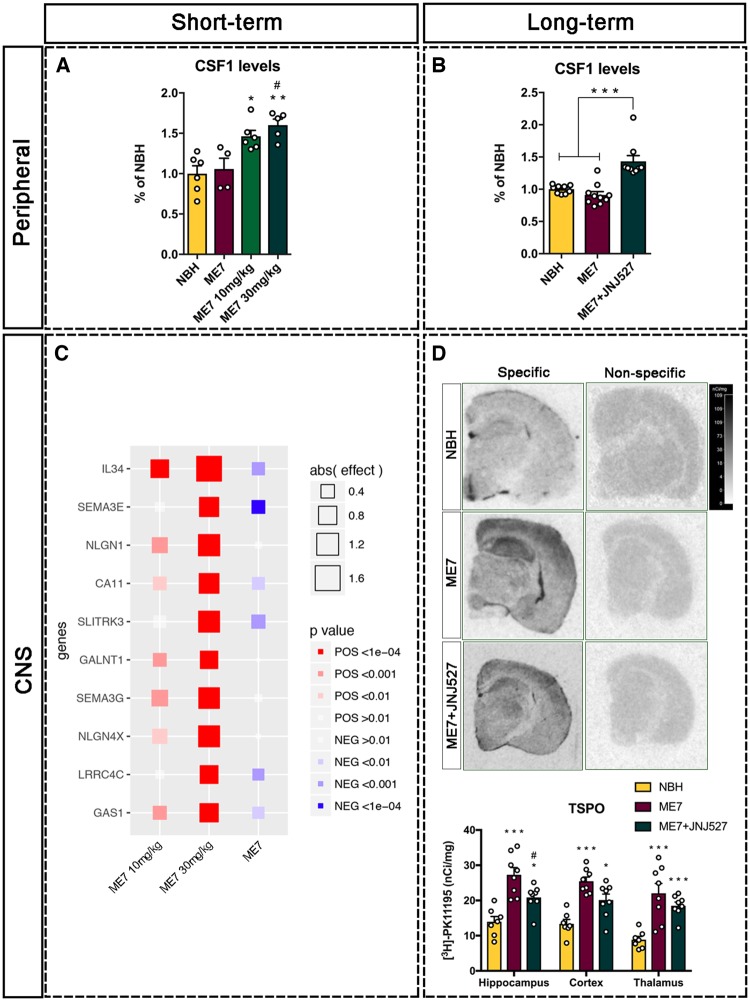
**Target engagement and efficacy readouts for clinical testing of JNJ-527.** (**A** and **B**) Quantification of CSF1 in plasma was used as a measure of peripheral target engagement. (**A**) Short-term (5 days) administration of JNJ-527 administration induced an increase of plasma CSF1 (NBH *n = *8, ME7+JNJ-527 10 mg/kg *n = *6, ME7+JNJ-527 30 mg/kg). (**B**) Long-term JNJ-527 administration (4 weeks) showed similar increments in plasma CSF1, reflecting consistent and long lasting target engagement in the systemic compartment (NBH *n = *10, ME7 *n = *8, ME7+JNJ-527 *n = *9). (**C**) Assessment of target engagement by JNJ-527 in the CNS. CSF high dimensionality proteomics with aptamer capture arrays showed a dose dependent alteration in IL-34 and SEMA3E (FDR *P < *9.19 × 10^−13^), among 67 other proteins (NBH *n = *29, ME7 *n = *30, with each type receiving JNJ-527 doses: vehicle *n = *10; 10 mg/kg *n = *10; 30 mg/kg *n = *9, 10). (**D**) Analysis of long-term efficacy by TSPO imaging after long-term treatment with JNJ-527. TSPO autoradiography with [3H]-PK11195 performed in different brain regions showed a consistent increase of signal in ME7 versus NBH mice, and an attenuation after treatment with JNJ-527 (NBH *n = *10, ME7 *n = *8, ME7+JNJ-527 *n = *9). For all graphs, values are mean ± SEM. *P < 0.05, **P < 0.01, ***P < 0.001 versus NBH; ^#^*P < *0.05 versus ME7.

Given the suitability of the ME7-prion model to study the effect of JNJ-527 at both short- (5 days) and long-term (4 weeks), we explored a number of readouts with potential application in clinical studies. We determined the extent of JNJ-527 target engagement in the systemic compartment by measuring the levels of CSF1 in plasma, as it was reported previously that chronic blockade of CSF1R in humans results in a 6-fold increase in plasma CSF1 due to the lack of internalization by the receptor (Genovese *et al.*, 2015). Using the same experimental set up described above, we found that 5 days CSF1R blockade by JNJ-527 led to a 30–40% increase in the levels of CSF1 in ME7-prion (Fig. 3A). The same analysis performed on plasma samples of ME7-prion mice treated during 4 weeks also showed a significant increase of 30% in the plasma levels of CSF1 (Fig. 3B). This demonstrates successful and sustained peripheral target engagement of JNJ-527.

Next, we set out to determine target engagement and efficacy of JNJ-527 in the CNS. For assessing target engagement in the brain, we used high dimensionality proteomics with aptamer capture arrays to determine differential expression of multiple proteins in CSF from both ME7 and NBH mice subjected to 5 days treatment with increasing doses of JNJ-527. We detected a dose dependent increase in IL-34, and SEMA3E (FDR *P* < 9.19 × 10^−13^; Fig. 3C) and 67 other proteins (Supplementary Table 1). Interestingly, IL-34 is one of the ligands of CSF1R and its increase in response to JNJ-527 shows a clear effect of compound on its intended target in the brain. CSF1 was not determined in this assay because the human specific probes used were not cross-reactive with the murine CSF1. In addition, we explored protein-wide expression changes further by using pathway enrichment for our experimental comparisons. The most significantly enriched pathways in response to increasing doses of JNJ-527 included axon guidance (*P = *0.00019), cell adhesion (*P = *0.00452) and extracellular matrix-receptor interactions (*P = *0.00014) as well as complement and coagulation cascades (*P = *0.00175); the latter an important immune response pathway (Supplementary Table 1).

Finally, we assessed the long-term efficacy of JNJ-527 in the brain by performing autoradiography on tissue sections using the TSPO ligand ^3^H-PK11195. Mitochondrial TSPO (18-kDa translocator protein) is a widely used imaging biomarker of neuroinflammation in humans and animal models, and its increased expression is linked to microglial activation, although TSPO can also be expressed in other immune and endothelial cells in the CNS (Turkheimer *et al.*, 2015). We found that the TSPO signal was significantly increased (>100%) in the hippocampus, somatosensory cortex and thalamus of ME7 versus NBH mice, and this increase was attenuated after the treatment with JNJ-527 (Fig. 3D). The 30% reduction in TSPO signal observed in the hippocampus parallels the reduction of microglial numbers after long-term treatment with JNJ-527 (Fig. 2A). These data provide evidence for sustained engagement and efficacy of JNJ-527 in the brain.

### Tau phosphorylation and degeneration of motor neurons are associated with an inflammatory reaction in P301S mice

As we had demonstrated the anti-proliferative effect of JNJ-527 in ME7 mice, we investigated the potential impact of blockade on CSF1R in the P301S mouse model of tauopathy. Building on previous studies of this model (Allen *et al.*, 2002; Bellucci *et al.*, 2004; Hampton *et al.*, 2010), we performed a longitudinal analysis first to confirm the dynamics of tau phosphorylation, neurodegeneration and microglial activation in the spinal cord of P301S mice to define a suitable therapeutic window for intervention with JNJ-527. We confirmed accumulation of phosphorylated tau in P301S mice at different time points, analysed by different phosphorylated epitopes (AT8, AT100 and AT180). Interestingly, different phospho-epitopes displayed variable patterns of change during the disease process, with an earlier increase at AT100 epitopes from 12 weeks of age (Supplementary Fig. 4A). We studied the specific localization of AT8 and AT100 phosphorylated tau in L4–L5 segments of P301S mice spinal cord and found the first sign of phospho-tau accumulation in spinal motor neurons at 12 weeks of age, which then spread throughout all the anterior spinal horn by 20 weeks (Supplementary Fig. 4B). The pattern of increased tau phosphorylation paralleled a significant loss of motor neurons from 12 weeks of age that progresses up to a 40% loss at end stages of the disease (Supplementary Fig. 4C).

**Figure 4:**
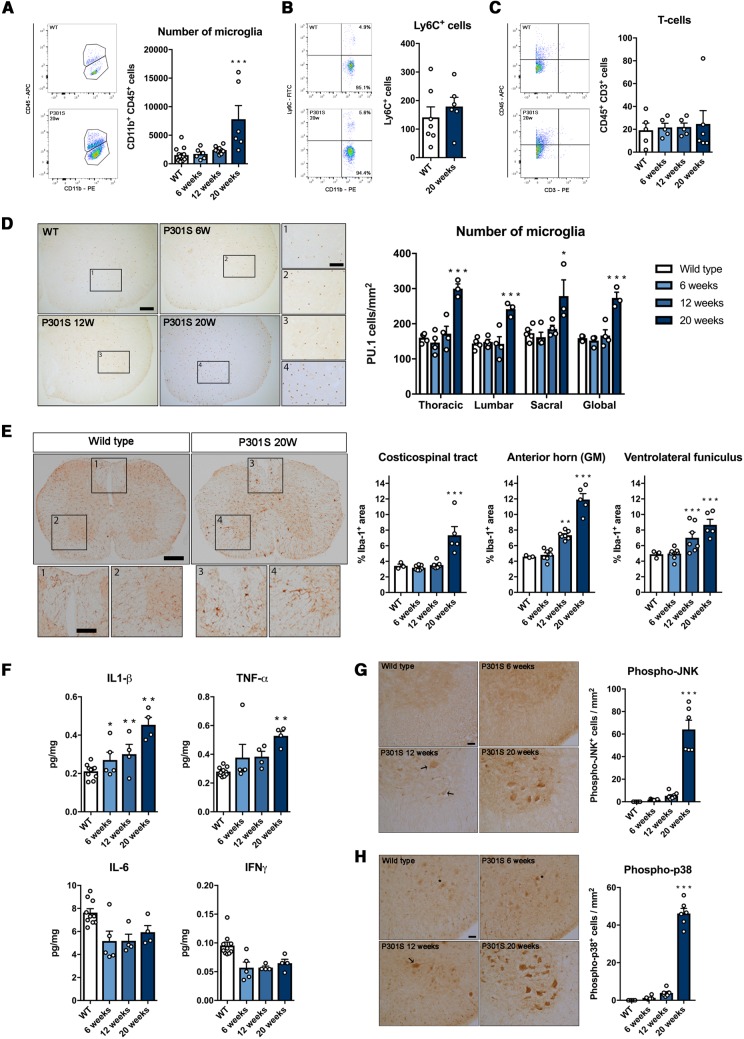
**Tau phosphorylation and degeneration of motor neurons is accompanied by an inflammatory reaction in the spinal cord of P301S mice.** (**A**) Significant increase in the number of microglia in the spinal cord of 20-week-old P301S mice, with no evidence of (**B**) infiltration of monocytes or (**C**) T cells [CD11b+ CD45+ cells, wild-type (WT)-20 weeks *n = *13, P301S-6 weeks *n = *6, P301S-12 weeks *n = *8, P301S-20 weeks *n = *6]. (**D**) The increase in the number of microglia is similar between different regions of the spinal cord. Scale bar = 100 μm. Higher magnification in boxes are shown in **1**–**4**. Scale bar = 25 μm (WT-20 weeks *n = *5, P301S-6weeks *n = *4, P301S-12weeks *n = *5, P301S-20 weeks *n = *4). (**E**) Morphological changes in microglia precede the increase in their number both in the white and grey matter of P301S spinal cord. Scale bar = 200 μm. Higher magnification in boxes are shown in 1–4. Scale bar, 25 μm (wild-type-20 weeks *n = *5, P301S-6weeks *n = *4, P301S-12weeks *n = *5, P301S-20 weeks *n = *4). (**F**) IL-1β and TNFα are significantly increased in the spinal cord of P301S mice, correlating with the degeneration of motor neurons (wild-type-20 weeks *n = *10, P301S-6 weeks *n = *5, P301S-12 weeks *n = *4, P301S-20 weeks *n = *4). (**G** and **H**) Activation of JNK (phospho-JNK) in the spinal cord of wild-type and P301S mice (wild-type-20 weeks *n = *5, P301S-6 weeks *n = *4, P301S-12 weeks *n = *5, P301S-20 weeks *n = *4). (**G**) Cytoplasmic accumulation of phospho-JNK in motor neurons starts by 12 weeks of age (arrows) and later affects both motor neurons and other smaller populations of neurons in the spinal cord. (**H**) Activation of p38 (phospho-p38) shows a similar pattern, with a translocation from the nucleus (asterisk) to the cytoplasm (arrows) of motor neurons of P301S mice from 12 weeks of age. Scale bar = 20 μm. Values are mean ± SEM. *P < 0.05, **P < 0.01 and *P < *0.001 versus wild-type.

An increased number of microglia is a hallmark of disease progression in mouse models of Alzheimer’s disease (Olmos-Alonso *et al.*, 2016), FTD (Clayton *et al.*, 2017), ALS (Martinez-Muriana *et al.*, 2016) and prion disease (Gomez-Nicola *et al.*, 2013). Similarly, in the P301S model, we quantified microglia in the spinal cord of P301S mice and found a consistent 2–3-fold increase in the number of cells by both flow cytometry (CD11b+ CD45+ cells) and in the density of cells by histology (PU.1 cells) (Fig. 4A and D). We also investigated a potential contribution of peripheral immune cells, and ruled out a significant influx of monocytes (CD45+ CD11b+ Ly6C+ cells) or T-cells (CD45+ CD3+ cells) infiltrating the spinal cord of these mice (Fig. 4B and C), and therefore we considered the totality of CD11b+ CD45+ cells as microglia. Interestingly, we did not observe any differential increase in microglia density across spinal cord regions, with a similar magnitude of change in thoracic, lumbar and sacral spinal segments in disease progression (Fig. 4D). Further histological analysis revealed an early increase in the Iba-1 staining area in the anterior regions of the spinal cord from 12 weeks of age, both in the grey matter and the ventrolateral funiculus (Fig. 4E). The changes in the microglial population were accompanied by a significantly increased expression of pro-inflammatory cytokines IL-1β and TNFα, the former being overexpressed from early pre-symptomatic stages (Fig. 4F), but not other cytokines (Supplementary Fig. 5). This suggests that microglia undergo phenotypic changes in response to neurodegeneration that are then followed by increased proliferation.

It has been shown previously that inflammatory cytokines, especially IL-1β, can contribute to tau phosphorylation by the activation of MAPK in neurons in hTau mice (Maphis *et al.*, 2015, 2016). Therefore, we explored the levels of activation of JNK (phospho-JNK) and p38 (phospho-p38) in spinal motor neurons of P301S mice, identified as those present in lamina IX with diameters larger than 20 μm, polygonal shape and prominent nucleoli. We found an age-dependent increase in the number of both phospho-JNK and phosho-p38 positive motor neurons in the L4-L5 segments of the spinal cord (Fig. 4G and H), which follows the same pattern as the increase of phosphorylated tau (Supplementary Fig. 4A and B). Interestingly, whereas in wild-type or early pre-symptomatic P301S mice (6 weeks) phospho-MAPK expression is localized in the nucleus of the cells (Fig. 4G and H), it then shifts to the cytoplasmic compartment from 12 weeks of age where MAPK could contribute to tau phosphorylation. Overall, our data suggest that inflammatory changes in the spinal cord parallel tau phosphorylation and degeneration of motor neurons, with first signs of alterations from 12 weeks of age.

### Blockade of CSF1R by JNJ-527 reduces microglial numbers and the expression of pro-inflammatory cytokines in P301S mice

**Figure 5:**
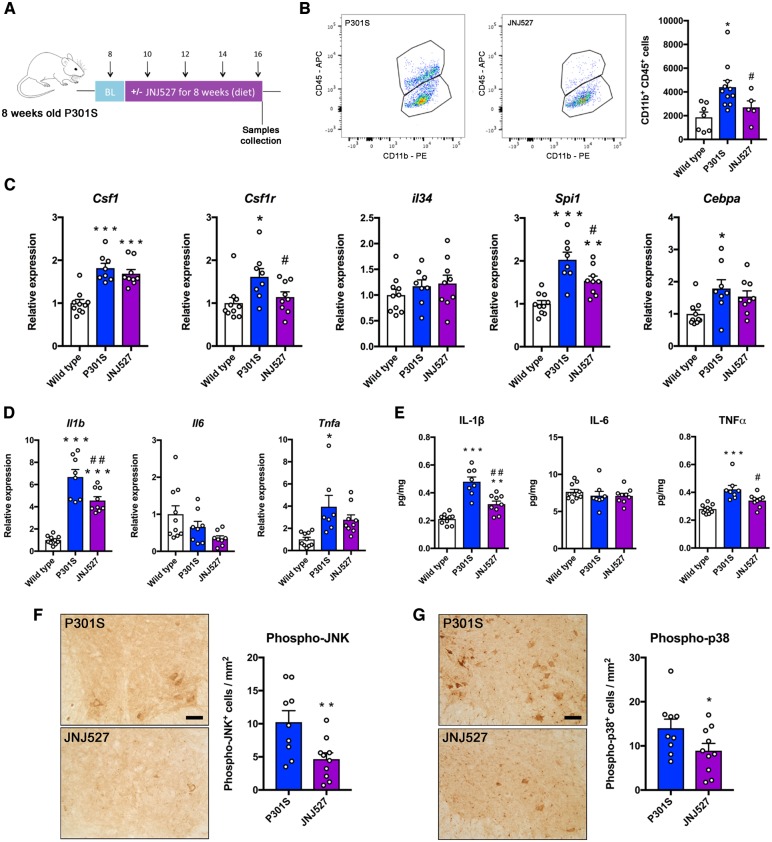
**JNJ-527 reduces microglial expansion and the expression of pro-inflammatory cytokines in P301S mice.** (**A**) Schematic representation of the experimental design used to test the efficacy of CSF1-R blockade in P301S mice. Animals were treated for 8 weeks with JNJ-527 at a dose of 30 mg/kg. (**B**) JNJ-527 treatment reduced the number of CD11b+ CD45+ cells in the spinal cord of P301S mice [wild-type (WT) *n = *7, P301S *n = *11, P301S+JNJ-527 *n = *6]. (**C**) JNJ-527 treated mice showed a significant reduction in the expression of genes associated with microglial proliferation (wild-type *n = *10, P301S *n = *8, P301S+JNJ-527 *n = *9). (**D** and **E**) The reduction in the number of microglia was correlated with a reduction of IL1-β and TNFα at (**D**) RNA and (**E**) protein level (wild-type *n = *10, P301S *n = *8, P301S+JNJ-527 *n = *9). (**F** and **G**) Diminished expression of inflammatory cytokines correlated with reduced activation of JNK and p38 in the cytoplasm of spinal motor neurons of 16 weeks old P301S mice (P301S *n = *9, P301S+JNJ-527 *n = *10). Scale bar = 50 μm. Values are mean ± SEM. *P < 0.05, **P < 0.01 and ***P < 0.001 versus P301S.

Since we had demonstrated that JNJ-527 was able to block microglial proliferation *in vivo*, we tested whether preventing microglial proliferation had an impact in the pathology of P301S tau transgenic mice. Given that we detected the first pathological signs in the spinal cord of P301S mice from 12 weeks of age, we performed a long-term preventive treatment from 8 to 16 weeks of age (Fig. 5A). Blockade of CSF1R in P301S mice led to a significant reduction in the number of cells, evidenced by 40% decrease in the number of microglial and macrophages (CD11b+ CD45+) in the lumbar spinal cord (Fig. 5B). The reduction in the number of microglia was accompanied by a significantly diminished expression of multiple genes controlling microglial proliferation, including *Csf1r* and *Spi1* (Pu.1) (Fig. 5C). These effects resembled those reported in other models of neurodegeneration including prion disease (Gomez-Nicola *et al.*, 2013) and mSOD1 ALS mice (Martinez-Muriana *et al.*, 2016) and while they may reflect a reduction in the number of microglia, we cannot exclude a diminished expression of these genes per cell. JNJ-527 also caused a significant reduction of the expression of IL1β and TNFα (Fig. 5D and E), without altering the levels of other cytokines (Supplementary Fig. 5). We then investigated whether the attenuation in the expression of inflammatory cytokines by JNJ-527 had any impact on the activation of JNK (phospho-JNK) and p38 (phospho-p38) in spinal motor neurons of P301S mice, which were identified by location, morphology and size: only polygonal cells with a diameter >20 μm and located in spinal lamina IX were counted. In both cases, we found a significant reduction of 60% and 40% in the density of motor neurons with cytoplasmic phospho-JNK or phospho-p38 expression in L4-L5 segments of the spinal cord, respectively (Fig. 5F and G). Our results show that JNJ-527 blocks microglial proliferation, attenuates the inflammatory response and modifies kinases activation in motor neurons in the P301S spinal cord.

### The reduction in the number of microglia impacts tau pathology and prevents motor neuron degeneration in P301S mice

**Figure 6:**
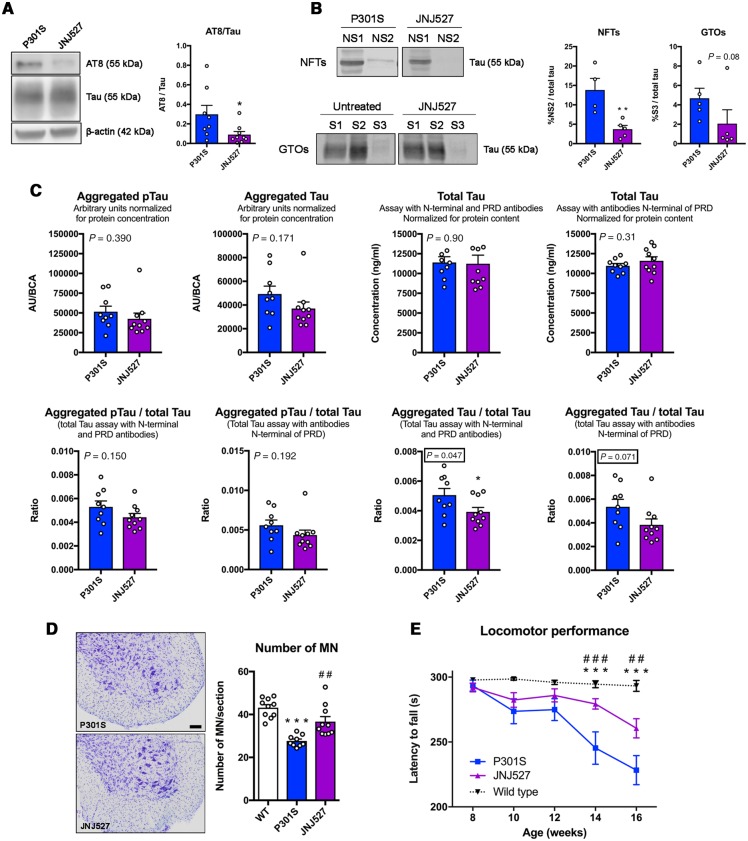
**Chronic blockade of CSF1R impacts tau pathology, prevents spinal motor neuron degeneration and improves motor function of P301S mice.** JNJ-527 administration reduced (**A**) tau phosphorylation (AT8) and (**B**) aggregation in the form of neurofibrillary tangles (neurofibrillary tangles, P301S *n = *4, P301S+JNJ-527 *n = *5) and globular tau oligomers (globular tau oligomers, P301S *n = *5, P301S+JNJ-527 *n = *5). Values are mean ± SEM. *P < 0.05, **P < 0.01 and ***P < 0.001 versus P301S. (**C**) JNJ-527 treatment induced a trend of tau aggregation reduction as measured by MSD and did not show an effect on total tau. Graphs are represented as mean ± SEM and the *P*-value from an unpaired *t*-test is indicated in the respective graphs, with near significant values outlined (P301S *n = *9, P301S+JNJ-527 *n = *10). (**D**) Quantification of motor neuron by Nissl staining showed increased neuronal preservation in L4–L5 spinal segments of 16-weeks-old P301S mice after JNJ-527 treatment (wild-type *n = *10, P301S *n = *9, P301S+JNJ-527 *n = *10), whereas (**E**) rotarod test showed improved motor function in JNJ-527 treated mice (wild-type *n = *20, P301S *n = *20, P301S+JNJ-527 *n = *21). Scale bar = 50 μm. For the neuronal survival and functional analysis, values are mean ± SEM. ***P < 0.001 versus wild type; ^##^*P < *0.01 and ^###^*P < *0.001 JNJ-527 versus P301S.

We then assessed whether blockade of microglial proliferation and modification of the inflammatory milieu by JNJ-527 had an impact on the neuropathology in the P301S spinal cord. Western blot analysis showed that treatment with JNJ-527 caused a significant reduction in the phosphorylation of tau at AT8 sites (Fig. 6A). We then performed protein fractionation to investigate the presence of granular tau oligomers or neurofibrillary tangles. Spinal cord homogenates were spun at speeds known to pellet either granular tau oligomers or larger tau fibrils (Cowan *et al.*, 2015), which were then dissolved in sarkosyl and termed the S3 and NS2 fractions, respectively. Mice treated with JNJ-527 showed a significant reduction in the insoluble NS2 fraction of larger tau fibrils and a trend towards a reduction in the insoluble NS3 fraction of globular tau oligomers (Fig. 6B). Additionally, we used a second biochemical method, an MSD immunoassay, to evaluate the amount of aggregated tau in the total homogenate of the spinal cord of the P301S mice. This aggregation assay consisted in a sandwich immunoassay with the same antibody used for coating as for detection. As such, both antibodies can only bind if multimers of tau are detected. Two aggregation assays were performed. One with the AT8 antibody to detect aggregates containing phospho-tau, and one with a total tau antibody binding in the proline riche domain to detect all aggregates, independent of their phosphorylation status. Furthermore, two sandwich immunoassays for total tau were performed. One with two non-competing antibodies with their epitope slightly N-terminal of the proline-rich domain and one with an antibody binding in the N-terminal region of tau and the other antibody binding in the proline-rich domain. Whereas both aggregation assays show no changes in the absolute values of aggregated tau in animals treated with JNJ-527, we found a tendency towards a reduction when corrected by total tau (Fig. 6C). Treatment with JNJ-527 also led to a significant preservation of motor neurons located in the spinal cord (Fig. 6D), and a significant improvement of motor function (Fig. 6E). Overall, these results demonstrate that the blockade of microglial proliferation by JNJ-527 might lead to attenuation of tau pathology and results in a reduction of neuronal cell death in P301S mice.

### JNJ-527 modifies the transcriptomic profile of the P301S mice spinal cord

Next, we sought to analyse the effect of JNJ-527 in the P301S mice at the transcriptomic level to determine whether the reduction in microglial numbers was also associated with a phenotypic change that could underpin the effects seen on tau pathology and neuronal preservation. In the first instance, both spinal cord and cortical samples were used to assess the impact of JNJ-527 (Supplementary Fig. 7A). Principal component analysis of the gene expression data showed that the first principal component (PC1) divided our samples by tissue and the second principal component (PC2) distinguished between P301S and wild type mice (Supplementary Fig. 7B). Similarly, the main branching point obtained by hierarchical clustering also divided the samples according to tissue (Supplementary Fig. 8). Taking into account that PC1 alone explained about 80% of the variance we decided to carry out differential expression analysis separately for the spinal cord and the cortex.

We compared the expression profile of wild-type versus P301S mice and found 900 (542 up- and 358 downregulated) and 944 (432 up- and 512 downregulated) differentially expressed genes in the spinal cord and cortex, respectively (FDR < 0.05). From these, 297 genes were differentially expressed in both tissues, which represents a higher overlap than that expected by chance (expected = 54.527, *P* < 1 × 10^−4^) and indicates a degree of consistency of the transcriptomic disease signal across tissues (Supplementary Fig. 7C and Supplementary Table 2).

**Figure 7:**
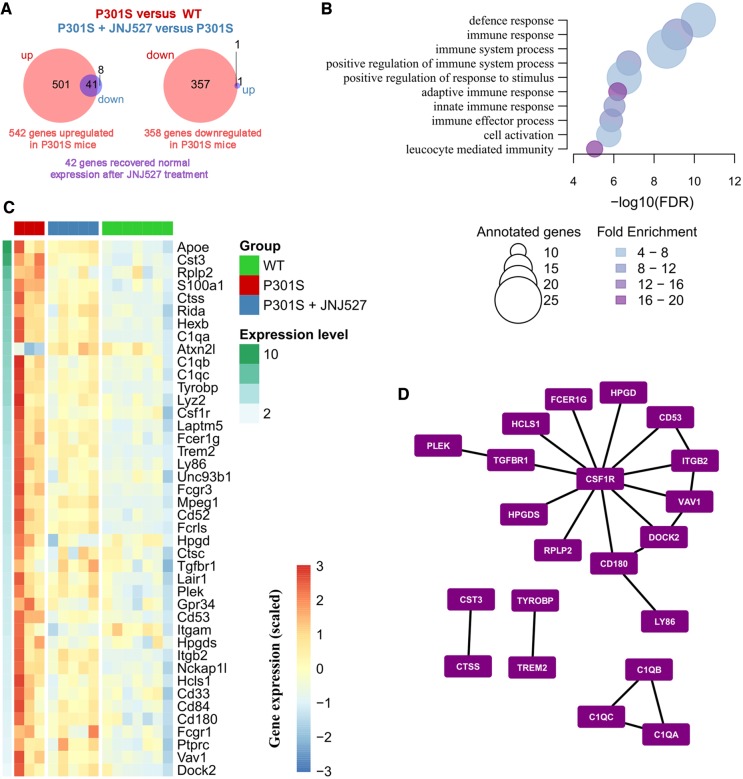
**Differentially expressed genes driven by JNJ-527 in the spinal cord of P301S mice.** (**A**) Venn diagrams showing opposing patterns of expression in disease (P301S versus wild-type) and treatment (P301S+JNJ-527 versus P301S) in the spinal cord (wild-type *n = *7, P301S *n = *3, P301S+JNJ-527 *n = *5). Briefly, 542 genes were upregulated (*left*) and 358 were downregulated (*right*) in the spinal cord of P301S mice. Of those genes, 42 recovered normal expression levels after chronic treatment with JNJ-527 (in purple). (**B**) FDR for top 10 biological processes overrepresented among the 41 genes upregulated in disease and downregulated with JNJ-527 treatment, the size of the circles reflects the number of genes annotated in each gene ontology (GO) term while the colour indicates the fold enrichment. (**C**) Heat map showing the gene expression patterns in the spinal cord samples for the overlapping genes shown in **A**, genes are ordered from *top* to *bottom* according to their average expression [log_2_ (TPM + 1)]. (**D**) From the aforementioned 41 genes, we selected those with a one-to-one orthologue in human and looked for known PPIs among them. The network shows the 22 known PPIs among the targeted genes, with CSF1R as a relevant hub.

Then, we focused on the effect of the JNJ-527 on the P301S tau mice. Strikingly, only one gene was differentially expressed in the cortex after the treatment, whereas 50 genes showed a significant response in the spinal cord (Fig. 7A and Supplementary Fig. 7C, FDR < 0.05), which reflects a selective impact of JNJ-527 in regions associated with increased microglial proliferation (Fig. 4A and D).

Further analysis of the expression of those 42 JNJ-527 responsive genes in the spinal cord showed that all of them had opposing directions in disease and treatment. The vast majority of them (41 genes of 49) were upregulated in disease and downregulated with treatment, with only the *Atx2nl* gene as the exception, which followed the opposite pattern, downregulated in disease and upregulated after treatment (Fig. 7A and B). By using a well-characterized set of cell-type specific markers from NeuroExpresso (Mancarci *et al.*, 2017), we looked for an overlap with known cell-type specific markers. Almost all genes showed a preference of expression in microglial cells. The only exceptions were *Rida* and *S100a1*, which showed the highest expression in Bergmann cells (Supplementary Fig. 9A), and *Apoe*, which showed very low expression in microglia in the healthy brain, but has been shown to be one of the most upregulated factors in disease-associated microglia (Keren-Shaul *et al.*, 2017; Krasemann *et al.*, 2017). We confirmed the cell-specific expression patterns of our set of 41 genes using two additional compilations from RNA-seq data (Supplementary Fig. 9B and C). Interestingly, a significant proportion of downregulated genes after JNJ-527 treatment are characteristic of the disease associated response that has been previously described in multiple models of disease, including P301S mice (Friedman *et al.*, 2018), such as *Apoe*, *Rplp2*, *Ctss*, *Tyrobp*, *Lyz2*, *Fcer1g*, *Trem2*, *Mpeg2*, *Cd52*, *Plek* and *Cd84* (for only two homeostatic microglial markers, *Hexb* and *Tgfb1*) (Keren-Shaul *et al.*, 2017; Krasemann *et al.*, 2017). Although we cannot exclude the possibility that this effect is due to a reduction in the density of microglia, it likely also reflect a shift in the transcriptomic profile of the remaining microglial cells, and raises interesting questions as to whether diminishing this disease-associated phenotype may have a beneficial effect in the context of neurodegeneration.

### Transcriptomic changes elicited by JNJ-527 involve immune networks with CSF1R as hub

Given the consistency of the treatment in reversing the transcriptomic profile of the disease, we looked for functional associations, first using pathway analysis and then using interaction analyses. Gene Ontology (GO) enrichment analysis revealed a strong immune related signature with the top ten enriched biological processes all related to immunity, and more than 50% of the genes (42) annotated to ‘immune system process’ (Fig. 7C).

Finally, we assessed whether the protein products of the 41 genes are known to interact with each other. Thus, combining diverse datasets we created an integrated PPI network for human and found 22 known PPIs among the 41 genes. The number of PPIs detected was higher than that expected by chance, as estimated by 10 000 random permutations (*P* < 0.0001). As expected, CSF1R was a hub in the PPI network participating in half of the known PPIs (Fig. 7D).

## Discussion

In this study we used two complementary animal models of protein misfolding neurodegenerative disease, with a prominent contribution of microglia, to validate the new selective CSF1R inhibitor JNJ-527 for the blockade of microglial proliferation and assess its efficacy in tau-induced neurodegeneration. We first used the ME7 prion model, which presents a rapid expansion of microglia that is at least partially driven by CSF1R (Gomez-Nicola *et al.*, 2013), and showed that JNJ-527 is brain penetrant, affects microglial proliferation in a dose dependent manner, and elicits changes in microglia that can be detected by clinically relevant readouts, including TSPO PET ligand binding and CSF proteomics, which are highly translational and add significant value for the preparation of further clinical studies. We then moved to a more relevant model for tauopathies and Alzheimer’s disease and assessed the impact of CSF1R inhibition by JNJ-527 in P301S mice. We showed that blocking the expansion of microglia impacts microglial phenotype and the CNS inflammatory milieu, and may ameliorate tau pathology, attenuates neurodegeneration and results in functional improvement. Overall, our study establishes the potential for clinical application of the CSF1R inhibitor JNJ-527 in Alzheimer’s disease and other neurodegenerative disorders where neuroinflammation may play a role.

The importance of neuroinflammation and microglia in Alzheimer’s disease has been highlighted by multiple GWAS, which have identified a number of single polymorphisms associated with risk for development of Alzheimer’s disease in several immune related genes, including *TREM2*, *CD33*, *BIN1*, and *CR1* (Efthymiou and Goate, 2017). Therefore, understanding the role of microglia in the different aspects of Alzheimer’s disease seems crucial to develop successful strategies to tackle the disease. Among the multiple aspects of the inflammatory reaction occurring in the Alzheimer’s disease brain, several studies have highlighted the contribution of microglial proliferation as a significant pathogenic process (Spangenberg and Green, 2017). Either blockade of proliferation or partial ablation of microglia in APP/PS1 (Olmos-Alonso *et al.*, 2016), 3xTg-AD (Dagher *et al.*, 2015) and 5xTgAD (Spangenberg *et al.*, 2016; Sosna *et al.*, 2018) models resulted in prevention of synaptic pathology and attenuation of cognitive deficits. However, while these studies have focused on amyloid-β pathology, the link between neuroinflammation and tau pathology, the other major proteinopathy in the Alzheimer’s disease brain, or tau-mediated neurodegeneration remains to be elucidated.

Previous studies have shown that induction of microglial activation by systemic administration of lipopolysaccharide or genetic deletion of *Cx3cr1* enhances tau phosphorylation/aggregation in both wild-type (Bhaskar *et al.*, 2010) and hTau transgenic mice (Maphis *et al.*, 2015, 2016). In addition, genetic deletion of *Cx3cr1* in hTau mice induces or increases neuronal loss and cognitive deficits (Maphis *et al.*, 2015). Abnormal activation of JNK and p38 has also been reported in motor neurons of P301S mice (Allen *et al.*, 2002; Bellucci *et al.*, 2004) and inflammatory cytokines, especially IL-1β, can contribute to tau phosphorylation via the activation of MAPK in neurons in hTau mice (Bhaskar *et al.*, 2010; Maphis *et al.*, 2015, 2016). Microglia have been also suggested to play a role in tau propagation across the brain by transferring phosphorylated tau from neuron to neuron possibly via exosomes (Asai *et al.*, 2015). Nevertheless, it is still uncertain whether microglia have a predominant effect on tau-related pathology and whether its modulation may alter disease course. Here, we show that inhibition of microglial proliferation in P301S mice resulted in a modification of the microglial phenotype, a reduction of inflammatory cytokines and an attenuation of MAPK activity in the cytoplasm of P301S spinal motor neurons, potentially slowing down tau aggregation, and preventing neurodegeneration and motor deficits. Although the blockade of microglial proliferation seems to have an impact on tau aggregation, similar strategies have failed to modify the amyloid-β deposition in APP/PS1 (Olmos-Alonso *et al.*, 2016), 3xTg-AD (Dagher *et al.*, 2015) and 5xTg-AD (Spangenberg *et al.*, 2016; Sosna *et al.*, 2018). This may point to a fundamentally different involvement of microglia in pathological processes driven by amyloid-β or tau, but recent work from Sosna *et al.* (2018) also suggests that this might just be the result of the selected timing for treatment, since the early ablation of microglia in 5xTg-AD resulted in a reduction of soluble and insoluble forms of amyloid-β. Interestingly, it has also been shown that IL-1β can increase APP synthesis in human cells by post-transcriptional regulation (without altering *APP* mRNA levels) (Rogers *et al.*, 1999), and therefore the discrepancy in the results obtained from amyloid-β mouse models may also be explained by a disease stage-dependent involvement of inflammatory cytokines. Here, we provide compelling evidence that activated microglia play a deleterious role within the context of tau-related diseases and that, in P301S mice, this effect may be at least partially mediated by the production of inflammatory cytokines which have an impact on the physiology of neurons. Partial depletion of microglia has been also recently tested in aged Tg2510 but failed to modify tau pathology (Bennett *et al.*, 2018). Whereas this discrepancy is more likely because of the particular therapeutic window selected, as the partial microglial depletion was induced when full tau pathology was already established in the brain of these mice, it is also plausible that differences are the result of the nature of the tyrosine kinase inhibitor used, PLX3397, which shows more selectivity for KIT than CSF1R (20 nM, 10 nM and 160 nM for CSF1R, KIT and FLT3, respectively) (DeNardo *et al.*, 2011).

Recent transcriptomics performed on 5xTg-AD (Keren-Shaul *et al.*, 2017), APP/PS1 (Krasemann *et al.*, 2017) and P301S (Friedman *et al.*, 2018) mice has shed some light on the transcriptional signature of disease associated microglia and brain resident macrophages, in which there is a switch between a homeostatic signature characterized by the expression of genes such as *Tmem119*, *P2ry12*, *Cx3cr1*, and *Sall1* to a pathogenic phenotype, which upregulates a very specific set of genes including *Trem2*, *Apoe*, *Csf1*, *Itgax*, *Cst7*, *Clec7a*, with *Trem2* and especially *Apoe* as major drivers of the phenotypic changes (Keren-Shaul *et al.*, 2017; Krasemann *et al.*, 2017). To gain in-depth knowledge about the potential mechanism underlying the positive impact of CSF1R inhibition by JNJ-527, we performed a transcriptomic analysis of the spinal cord of treated versus untreated P301S mice. We found that the treatment reduced the expression of several disease-associated genes including *Apoe*, *Rplp2*, *Ctss*, *Tyrobp*, *Lyz2*, *Fcer1g*, *Trem2*, *Mpeg2*, *Cd52*, *Plek* and *CD84* (Keren-Shaul *et al.*, 2017; Krasemann *et al.*, 2017; Friedman *et al.*, 2018). Although this may reflect a shift in the phenotype of the remaining microglia, we cannot exclude that it is due to a reduction in the density of cells. However, our findings suggest that modifying the microglial disease-associated phenotype may produce a beneficial impact in the context of brain disease.

Interestingly, from a functional perspective, both gene products that might activate microglia through their ITAM domains (*Tyrobp*, *Trem2*, *Fcer1g*, *Fcgr3*, *Fcrls*, *Fcrg1*) and genes that inhibit microglia activation through ITIM domains are downregulated after JNJ-527 treatment. This may reflect the duality of detrimental and protective roles that microglia can acquire in the context of neurodegeneration, which may also play a role in the beneficial effects we observed in terms of tau pathology and neuronal protection. Precisely how the shift in gene expression is reflected in the microglia secretome to impact on tau-mediated pathology remains to be established.

An important and often omitted aspect of preclinical research suggesting new therapeutic strategies for neurodegenerative diseases, is the use of specific tools that can be transferred to human patients. JNJ-527 is a clinically characterized compound that showed safety and tolerability when administered to humans (Genovese *et al.*, 2015; Von Tresckow *et al.*, 2015). We showed that JNJ-527 is a CNS-penetrant CSF1R inhibitor and established an optimal dose for preclinical applications, and estimate an efficacious dose for further clinical investigations in neurodegenerative disease. We also showed that measuring plasma levels of CSF1 is a consistent readout for both short- and long-term peripheral target engagement by JNJ-527 (Genovese *et al.*, 2015).

We then performed a set of experiments to determine the target engagement of JNJ-527 in the CNS. We first conducted TSPO autoradiography, as a precursor to the potential *in vivo* TSPO PET imaging in humans. TSPO is elevated in activated microglia of the CNS but also in astrocytes, endothelial cells, macrophages and monocytes in response to a variety of insults, as well as neurodegenerative diseases (Turkheimer *et al.*, 2015). TSPO is one of the few available imaging biomarkers to detect inflammation in the living human brain. We report for the first time an increase in TSPO binding in the ME7 mouse prion model. Importantly, TSPO binding was sensitive to the inhibition of CSF1R in the ME7 mouse model after JNJ-527 chronic treatment, showing a similar magnitude of change to the histological and flow cytometry analyses of microglia, and therefore showing promise as a potential clinical measure of treatment efficacy. We also used high dimensionality proteomics to determine differential expression of proteins in CSF upon administration of JNJ-527. Interestingly, we found a dose dependent increase in IL-34, which is one of the ligands of CSF1R in the CNS. This parallels the behaviour of CSF1 in plasma and represents invaluable evidence of target engagement within the CNS with potential for direct application in clinical studies.

Overall, this study shows that inhibition of microglial proliferation and modification of the inflammatory milieu is able to modify the disease trajectory in the P301S model of tau-induced neurodegeneration. These findings have clear relevance for Alzheimer’s disease, where tau is a major player, but also other tauopathies including multiple forms of FTD, and works towards providing a solid base for future testing of JNJ-527 in the clinic.

## Supplementary Material

awz241_Supplementary_Data

## Abbreviations

NBH: normal brain homogenate
PPI: protein–protein interaction

## Appendix 1

### NIMA (Part) Consortium members

Full details are provided in the Supplementary material. Asterisk indicates a former consortium member.

Edward T. Bullmore, Junaid Bhatti, Samuel J. Chamberlain, Marta M. Correia, Anna L. Crofts, Amber Dickinson,* Andrew C. Foster,* Manfred G. Kitzbichler, Clare Knight,* Mary-Ellen Lynall, Christina Maurice, Ciara O’Donnell, Linda J. Pointon, Peter St George Hyslop, Lorinda Turner, Petra Vertes, Barry Widmer, Guy B. Williams, B. Paul Morgan, Claire A. Leckey, Angharad R. Morgan,* Caroline O’Hagan,* Samuel Touchard, Jonathan Cavanagh, Catherine Deith,* Scott Farmer, John McClean, Alison McColl, Andrew McPherson,* Paul Scouller,* Murray Sutherland, H.W.G.M. (Erik) Boddeke, Jill C. Richardson, Shahid Khan, Phil Murphy, Christine A. Parker, Jai Patel, Declan Jones, Peter de Boer, John Kemp, Wayne C. Drevets, Jeffrey S. Nye (deceased), Gayle Wittenberg, John Isaac, Anindya Bhattacharya, Nick Carruthers, Hartmuth Kolb, Carmine M. Pariante, Federico Turkheimer, Gareth J. Barker, Heidi Byrom, Diana Cash, Annamaria Cattaneo, Antony Gee, Caitlin Hastings, Nicole Mariani, Anna McLaughlin, Valeria Mondelli, Maria Nettis, Naghmeh Nikkheslat, Karen Randall, Hannah Sheridan,* Camilla Simmons, Nisha Singh, Victoria Van Loo,* Marta Vicente-Rodriguez, Tobias C. Wood, Courtney Worrell,* Zuzanna Zajkowska,* Niels Plath, Jan Egebjerg, Hans Eriksson, Francois Gastambide, Karen Husted Adams, Ross Jeggo,* Christian Thomsen, Jan Torleif Pederson, Brian Campbell,* Thomas Möller,* Bob Nelson,* Stevin Zorn,* Jason O’Connor, Mary Jane Attenburrow, Alison Baird, Jithen Benjamin, Stuart Clare, Philip Cowen, I-Shu (Dante) Huang, Samuel Hurley,* Helen Jones, Simon Lovestone, Francisca Mada,* Alejo Nevado-Holgado, Akintayo Oladejo,* Elena Ribe, Katy Smith, Anviti Vyas,* Zoe Hughes,* Rita Balice-Gordon,* James Duerr,* Justin R. Piro,* Jonathan Sporn,* V. Hugh Perry (PI), Madeleine Cleal,* Gemma Fryatt, Diego Gomez-Nicola, Renzo Mancuso, Richard Reynolds, Neil A. Harrison, Mara Cercignani, Charlotte L. Clarke, Elizabeth Hoskins,* Charmaine Kohn,* Rosemary Murray,* Lauren Wilcock, Dominika Wlazly, Howard Mount.
