# Supplementary material for: CSF1R inhibitor JNJ-40346527 attenuates microglial proliferation and neurodegeneration in P301S mice
Source: Brain. 2019 Aug 26;142(10):3243–64. doi: 10.1093/brain/awz241 (PMC6794948; doi:10.1093/brain/awz241)
Supplement: awz241_Supplementary_Data [file awz241_supplementary_data.zip › awz241-Suppl_data/Supplementary_Data3.pdf]

NIMA (Part 1) Consortium members

Cambridge

Edward T. Bullmore (MD, PI, EC)[1,2,11], Junaid Bhatti[1], Samuel J. Chamberlain[1,2], Marta M. Correia[1,12], Anna L. Crofts[1], Amber Dickinson*, Andrew C. Foster*, Manfred G. Kitzbichler[1], Clare Knight*, Mary-Ellen Lynall[1], Christina Maurice[1], Ciara O'Donnell[1], Linda J. Pointon[1], Peter St George Hyslop[1,13,14], Lorinda Turner[31], Petra Vertes[1], Barry Widmer[1], Guy B. Williams[1,14]

Cardiff

B. Paul Morgan (PI)[15], Claire A. Leckey[15], Angharad R. Morgan*, Caroline O'Hagan*, Samuel Touchard[15]

Glasgow

Jonathan Cavanagh (PI, EC)[3], Catherine Deith*, Scott Farmer[16], John McClean[16], Alison McColl[3], Andrew McPherson*, Paul Scouller*, Murray Sutherland[16]

Independent advisor

H.W.G.M. (Erik) Boddeke (EC)[17]

GSK

Jill C. Richardson (EC)[18], Shahid Khan[11], Phil Murphy[19], Christine A. Parker[19], Jai Patel[11]

Janssen

Declan Jones (EC)[6], Peter de Boer[4], John Kemp[4], Wayne C. Drevets[6], Jeffrey S. Nye (deceased), Gayle Wittenberg[6], John Isaac[6], Anindya Bhattacharya[6], Nick Carruthers[6], Hartmuth Kolb[6]

Kings College London

Carmine M. Pariante (PI)[10], Federico Turkheimer (PI)[20], Gareth J. Barker[20], Heidi Byrom[10], Diana Cash[20], Annamaria Cattaneo[10], Antony Gee[20], Caitlin Hastings[10], Nicole Mariani[10], Anna McLaughlin[10], Valeria Mondelli[10], Maria Nettis[10], Naghmeh Nikkheslat[10], Karen Randall[20], Hannah Sheridan*, Camilla Simmons[20], Nisha Singh[20], Victoria Van Loo*, Marta Vicente-Rodriguez[20], Tobias C. Wood[20], Courtney Worrell*, Zuzanna Zajkowska*

Lundbeck

Niels Plath (EC)[21], Jan Egebjerg[21], Hans Eriksson[21], Francois Gastambide[21], Karen Husted Adams[21], Ross Jeggo*, Christian Thomsen[21], Jan Torleif Pederson[21], Brian Campbell*, Thomas Möller*, Bob Nelson*, Stevin Zorn*

University of Texas (sub-contracted to Lundbeck)
Jason O'Connor[22]

Oxford
Mary Jane Attenburrow (PI)[7,23], Alison Baird, Jithen Benjamin[23], Stuart Clare[25], Philip Cowen[7], I-Shu (Dante) Huang[24], Samuel Hurley*, Helen Jones[23], Simon Lovestone[7],(AD, PI, EC) Francisca Mada*, Alejo Nevado-Holgado[7], Akintayo Oladejo*, Elena Ribe[7], Katy Smith[23], Anviti Vyas*

Pfizer
Zoe Hughes*, Rita Balice-Gordon*, James Duerr*, Justin R. Piro*, Jonathan Sporn*

Southampton
V. Hugh Perry (PI)[27], Madeleine Cleal*, Gemma Fryatt[27], Diego Gomez-Nicola[27], Renzo Mancuso[32], Richard Reynolds[27]

Sussex
Neil A. Harrison (PI, EC)[28], Mara Cercignani[28], Charlotte L. Clarke[28], Elizabeth Hoskins*, Charmaine Kohn*, Rosemary Murray*, Lauren Wilcock[29], Dominika Wlazly[30]

University of Toronto (sub-contracted to Cambridge)
Howard Mount[13]

MD = Mood disorder workpackages lead
AD = Alzheimer's disease workpackages lead
PI = Principal Investigator
EC = Executive committee member

[1] Department of Psychiatry, School of Clinical Medicine, University of Cambridge, CB2 0SZ, UK
[2] Cambridgeshire and Peterborough NHS Foundation Trust, Cambridge, CB21 5EF, UK
[3] Sackler Centre, Institute of Health & Wellbeing, University of Glasgow, Sir Graeme Davies Building , Glasgow, G12 8TA, UK
[4] Neuroscience, Janssen Research & Development, Janssen Pharmaceutica NV, Turnhoutseweg 30, B-2340, Beerse, Belgium
[5] The Maurice Wohl Clinical Neuroscience Institute, Cutcombe Road, London, SE5 9RT, UK
[6] Neuroscience, Janssen Research & Development, LLC, Titusville, NJ, 08560, USA
[7] Department of Psychiatry, University of Oxford, Warneford Hospital, Oxford, OX3 7JX, UK

[8] Brighton & Sussex Medical School, University of Sussex, Brighton, BN1 9RR, UK
[9] Sussex Partnership NHS Foundation Trust, Swandean, BN13 3EP, UK
[10] Kings College London, Institute of Psychiatry, Psychology and Neuroscience, Department of Psychological Medicine, London, SE5 9RT, UK
[11] Immuno-Psychiatry, Immuno-Inflammation Therapeutic Area Unit, GlaxoSmithKline R&D, Stevenage SG1 2NY, UK
[12] MRC Cognition and Brain Sciences Unit, 15 Chaucer Road, Cambridge CB2 7EF, UK
[13] Tanz Centre for Research in Neurodegenerative Diseases, 60 Leonard Avenue, Toronto, ON M5T 2S8 Canada
[14] Department of Clinical Neurosciences, University of Cambridge, CB2 0SZ, UK
[15] Cardiff University, Cardiff CF10 3AT, UK
[16] NHS Greater Glasgow and Clyde, 1055 Great Western Rd, Glasgow G12 0XH, UK
[17] University of Groningen, 9712 CP Groningen, Netherlands
[18] Neurosciences Virtual PoC DPU, GlaxoSmithKline R&D, Stevenage SG1 2NY, UK
[19] Experimental Medicine Imaging, GlaxoSmithKline R&D, Stevenage SG1 2NY, UK
[20] King's College London, Department of Neuroimaging Sciences, Institute of Psychiatry, Psychology & Neuroscience, De Crespigny Park, London SE5 8AF, UK
[21] H. Lundbeck A/S Ottiliavej 9, 2500, Valby, Denmark
[22] University of Texas Health Science Center at San Antonio, 7703 Floyd Curl Dr, San Antonio, TX 78229, USA
[23] NIHR Oxford cognitive health Clinical Research Facility, Warneford Hospital, Oxford, OX3 7JX, UK
[24] The Kennedy Institute of Rheumatology, Roosevelt Dr, Oxford OX3 7FY, UK
[25] Oxford Centre for Functional MRI of the Brain, John Radcliffe Hospital, Oxford OX3 9DU, UK
[26] Pfizer, Inc, 1 Portland Street, Cambridge MA, USA
[27] Centre for Biological Sciences, University of Southampton, Southampton, UK
[28] Clinical Imaging Sciences Centre (CISC), University of Sussex, Brighton, BN1 9RR, UK
[29] Sussex Partnership NHS Foundation Trust, Nevill Avenue, Hove BN3 7HZ, UK
[30] Brighton & Sussex University Hospitals NHS Trust, Brighton BN2 5BE, UK
[31] Department of Medicine, School of Clinical Medicine, University of Cambridge, CB2 0SZ, UK
[32] VIB-KU Leuven Center for Brain & Disease Research, Campus Gasthuisberg, Herestraat 49, bus 602, 3000 Leuven, Belgium

*Former consortium members
