# Supplementary material for: CSF1R inhibitor JNJ-40346527 attenuates microglial proliferation and neurodegeneration in P301S mice
Source: Brain. 2019 Aug 26;142(10):3243–64. doi: 10.1093/brain/awz241 (PMC6794948; doi:10.1093/brain/awz241)
Supplement: awz241_Supplementary_Data [file awz241_supplementary_data.zip › awz241-Suppl_data/awz241_Supplementary_Fig.pdf]

Supplementary material

A

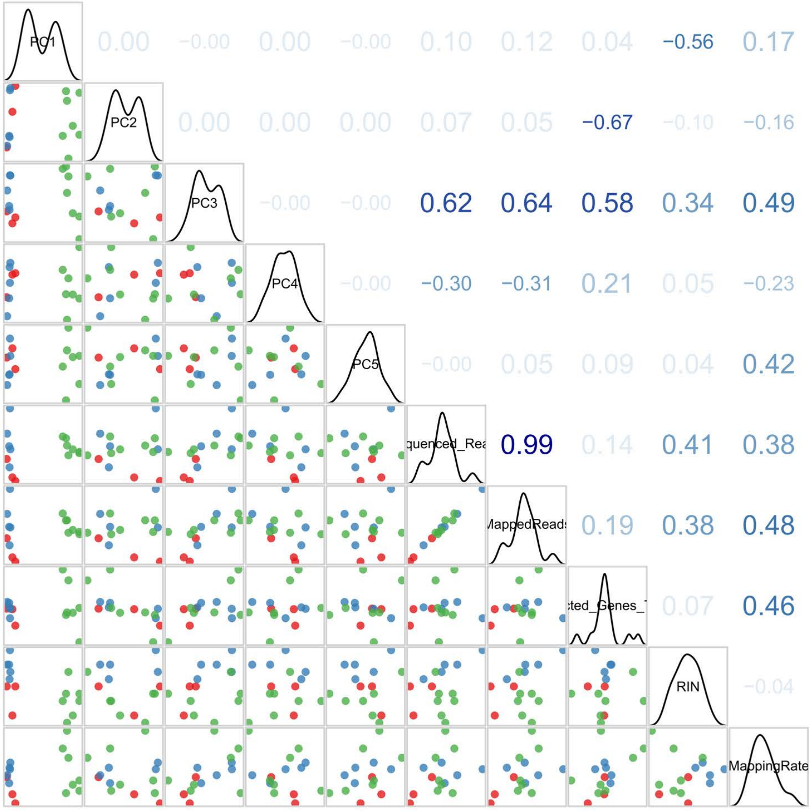
PC1
0.00 −0.00 0.00 −0.00 0.10 0.12 0.04 −0.56 0.17
PC2
0.00 0.00 0.00 0.07 0.05 −0.67 −0.10 −0.16
PC3
−0.00 −0.00 0.62 0.64 0.58 0.34 0.49
PC4
−0.00 −0.30 −0.31 0.21 0.05 −0.23
PC5
−0.00 0.05 0.09 0.04 0.42
quenced_Rea
0.99 0.14 0.41 0.38
MappedReads
0.19 0.38 0.48
cted_Genes_
0.07 0.46
RIN
−0.04
MappingRate

B

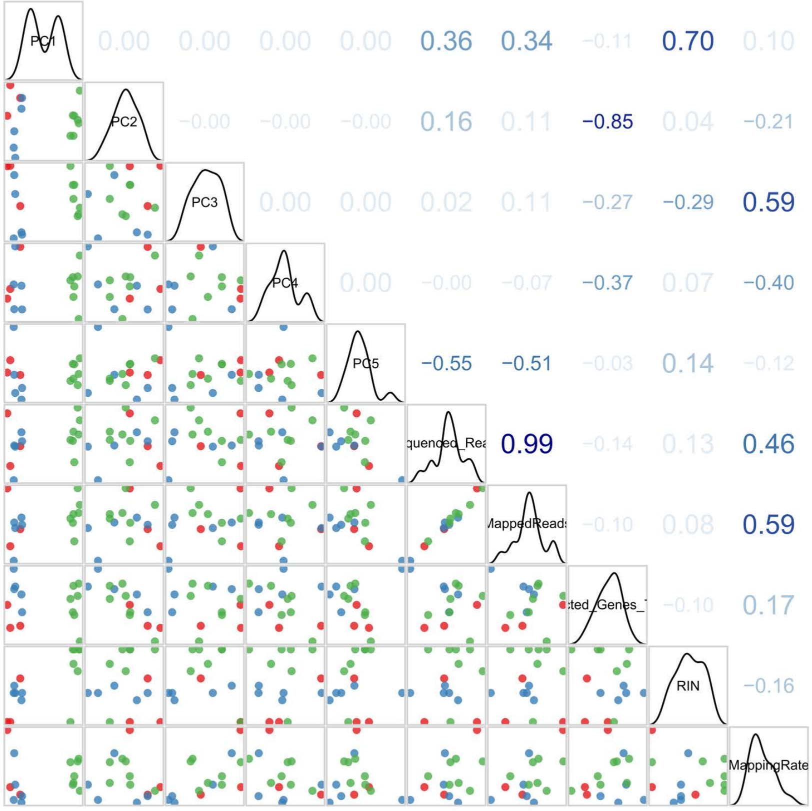
PC1
0.00 0.00 0.00 0.00 0.36 0.34 −0.11 0.70 0.10
PC2
−0.00 −0.00 −0.00 0.16 0.11 −0.85 0.04 −0.21
PC3
0.00 0.00 0.02 0.11 −0.27 −0.29 0.59
PC4
0.00 −0.00 −0.07 −0.37 0.07 −0.40
PC5
−0.55 −0.51 −0.03 0.14 −0.12
quenced_Rea
0.99 −0.14 0.13 0.46
MappedReads
−0.10 0.08 0.59
cted_Genes_
−0.10 0.17
RIN
−0.16
MappingRate

Figure S1. Selection of covariates for further differential gene expression analysis. Principal component analysis was done separately for (**A**) cortical and (**B**) spinal cord samples. The graph indicates in the upper triangle the correlation between the first 5 principal components, the number of sequenced reads, mapped reads, number of genes detected (at least 1 TPM), RIN score and mapping rate. In the lower triangle, the variables are plotted against each other separately for the (**A)** cortex and **(B)** the spinal cord. The colour of the points representing the samples match those in Fig. 7B, green (WT), red (P301S) and blue (P301S+JNJ-527).

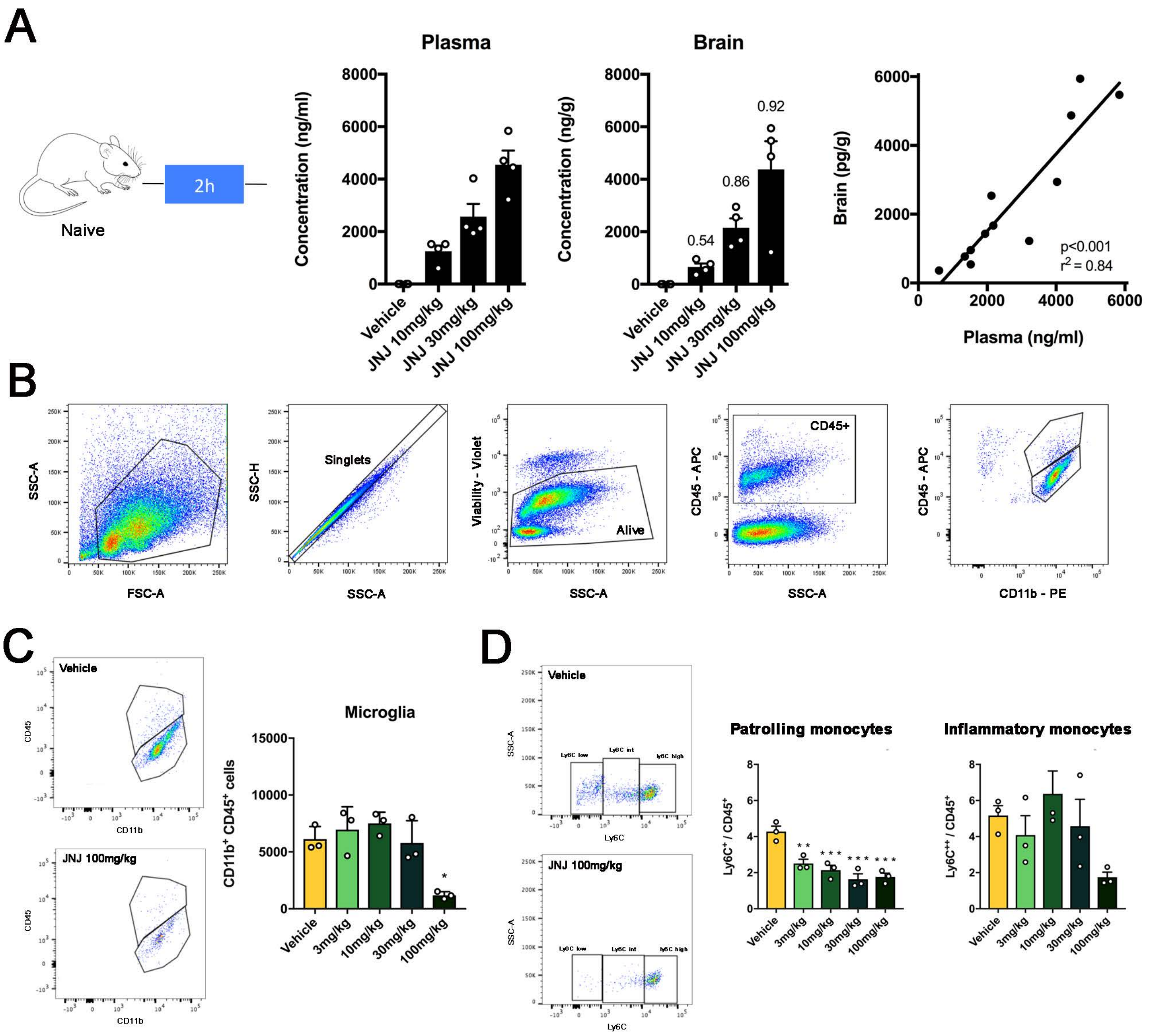

Figure S2. JNJ-527 penetrates into the brain and does not affect microglial survival at doses lower than 30mg/kg. (**A**) JNJ-527 brain penetrance was assessed in naïve mice after a single oral administration of JNJ-527 at 10, 30 and 100mg/kg (vehicle n=3, 10mg/kg n=3, 30mg/kg n=3, 100mg/kg n=3). Analysis by mass spectrometry showed a linear, dose dependent increase in the levels of the compound in both brain and plasma. Numbers above columns represent the tissue/plasma (T/P) ratio. (**B)** Representative flow cytometry plots showing the gating strategy used in this study. **(C, D**) Administration of JNJ-527 for 5 consecutive days (**C**) does not deplete microglia in doses below 30 mg/kg, whereas (**D**) significantly depletes blood non-classical monocytes (Ly6C+) without affecting classical monocytes population (Ly6C++) at all doses tested (vehicle n=3, 3mg/kg n=3, 10mg/kg n=3, 30mg/kg n=3, 100mg/kg n=3). Values are mean±SEM. *p<0.05, **p<0.01 and ***p<0.001 vs. vehicle.

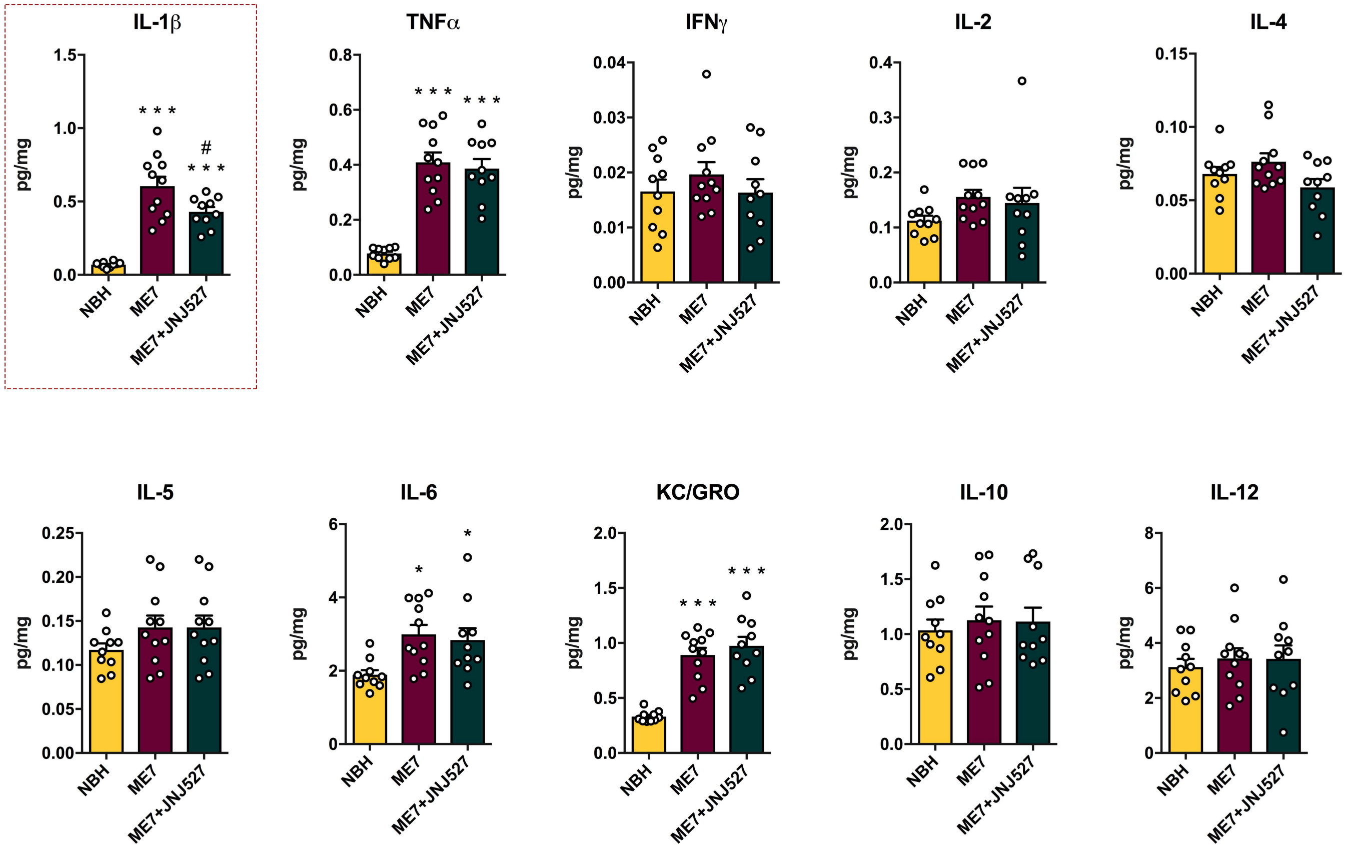

Figure S3. Extended cytokine panel in ME7 after long-term treatment (4 weeks) with JNJ-527. The red outline hightlights the cytokine already present in the main figures. For all quantifications, NBH n=10, ME7 n=8, ME7+JNJ-527 n=9. Values are mean±SEM. * $p<0.05$ *** $p<0.001$ vs. NBH; # $p<0.05$ vs. ME7.

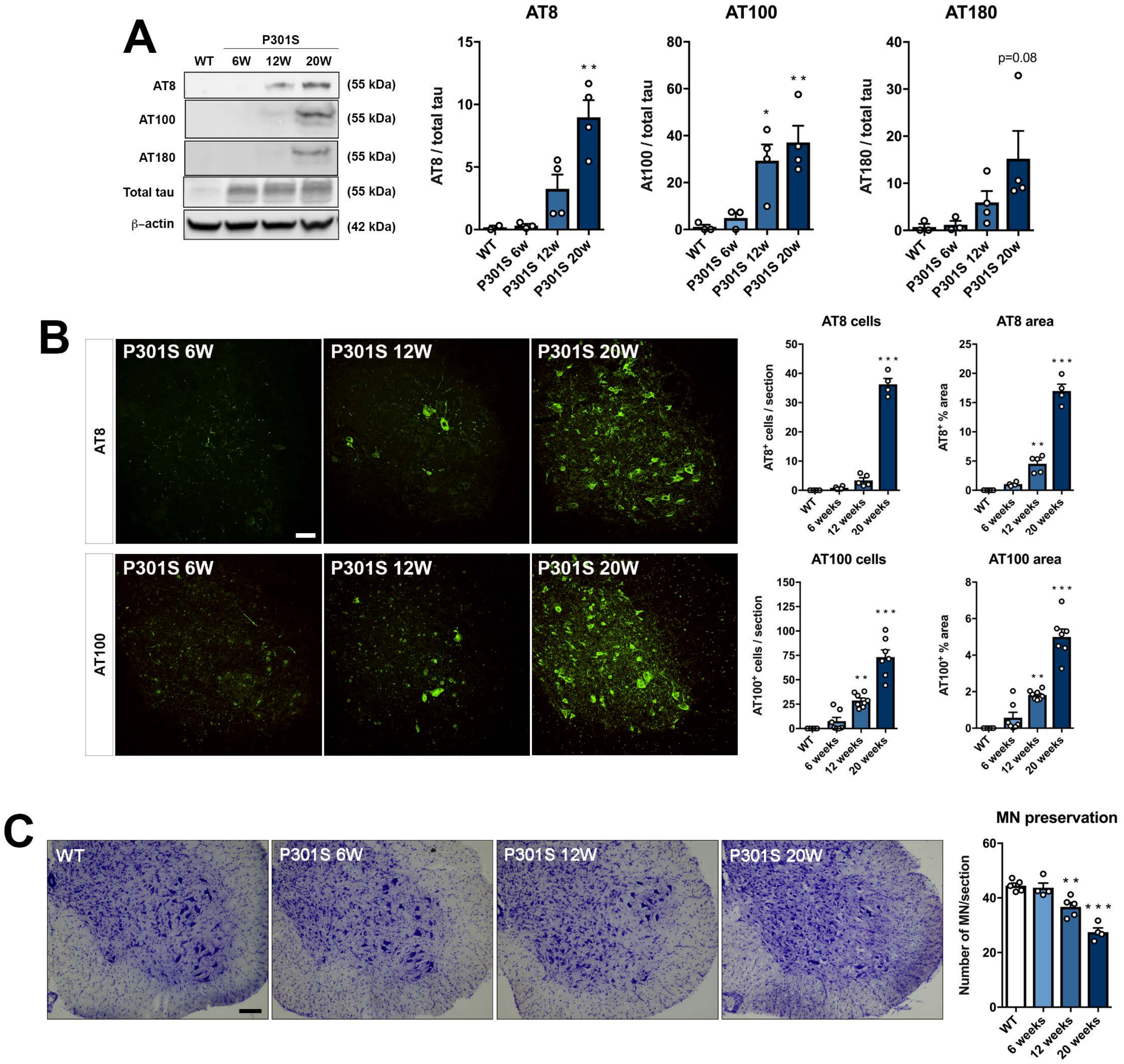

Figure S4. Age-dependent tau phosphorylation leads to motor neuron degeneration in the spinal cord of P301S mice. (**A**) Age dependent increase in tau phosphorylation (AT8, AT100 and AT180) in the lumbar spinal cord of P301S mice (WT-20 weeks n=3, P301S-6weeks n=4, P301S-12weeks n=5, P301S-20 weeks n=4). (**B**) Phosphorylated tau (AT8 and AT100) accumulates first in spinal motor neurons from 12 weeks of age and then spreads to other neuronal populations of the spinal cord (WT n=5, P301S-6weeks n=4, P301S-12weeks n=5, P301S-20 weeks n=4). Microphotographs show AT8 or AT100 in the anterior horn of L4-L5 spinal segments. (**C**) Tau phosphorylation leads to degeneration of spinal motor neurons (WT n=5, P301S-6weeks n=4, P301S-12weeks n=5, P301S-20 weeks n=4). Microphotographs show Nissl staining of the L4-L5 spinal cord, with arrows pointing to motor neuron cells bodies. Values are mean±SEM. * p<0.05, ** p<0.01 and p<0.001 vs. wild type.

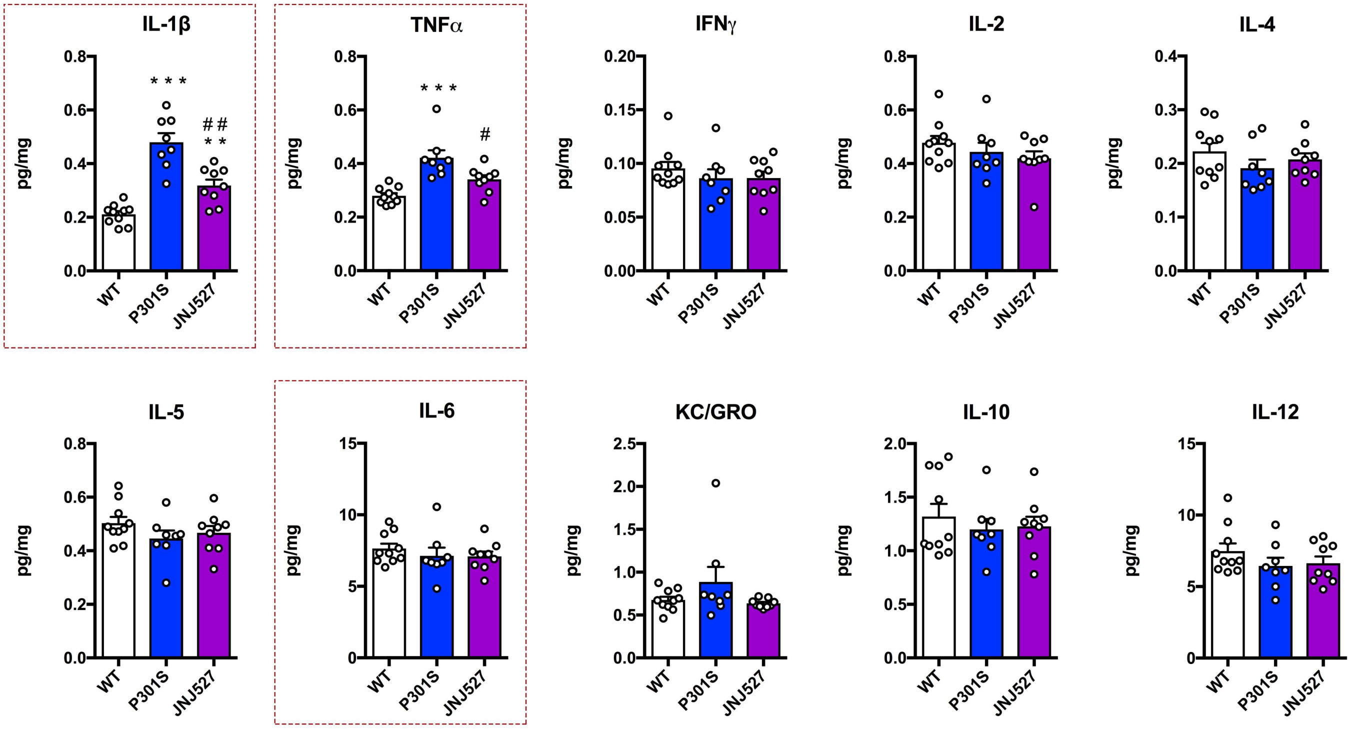

Figure S5. Extended cytokine panel in P301S after long-term (8 weeks) treatment with JNJ-527. The red outline boxes highlight the cytokines already present in the main figures. For all quantifications, P301S n=8, P301S+JNJ-527 n=9.

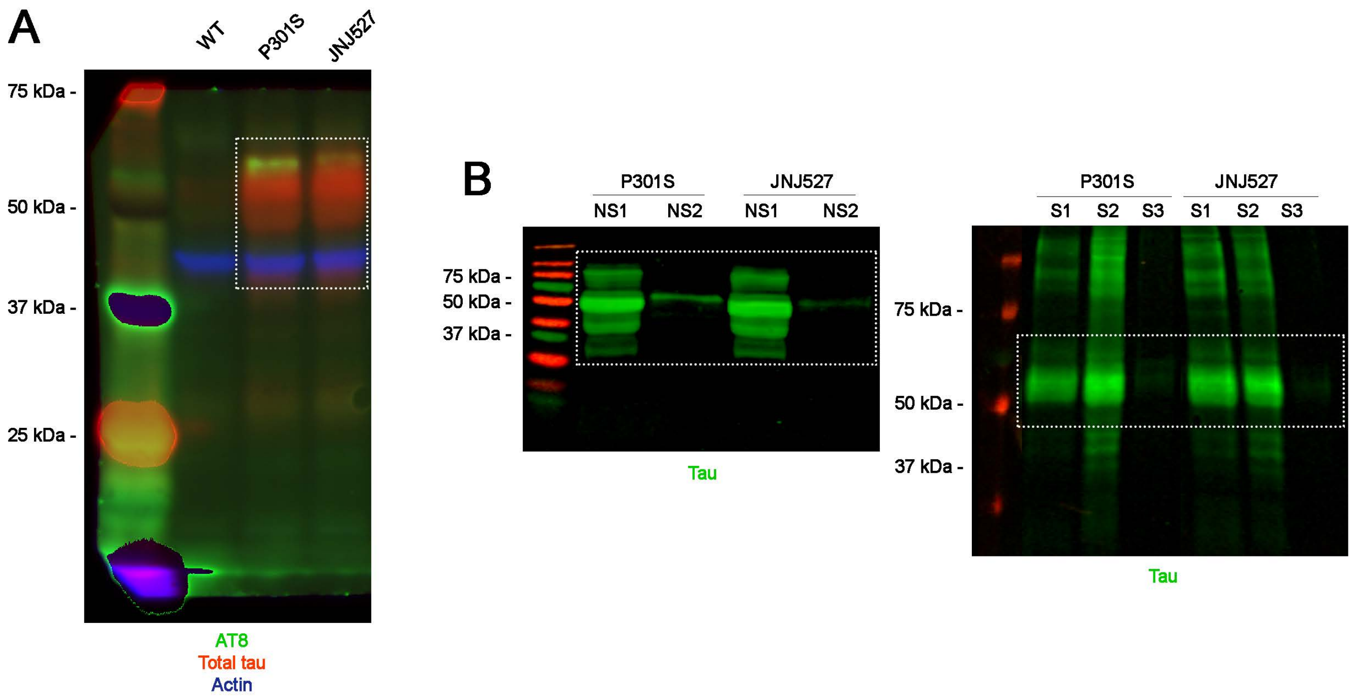

Figure S6. Full blots from Fig. 6A, B. The boxes highlight the part of the image cropped for Fig. 6.

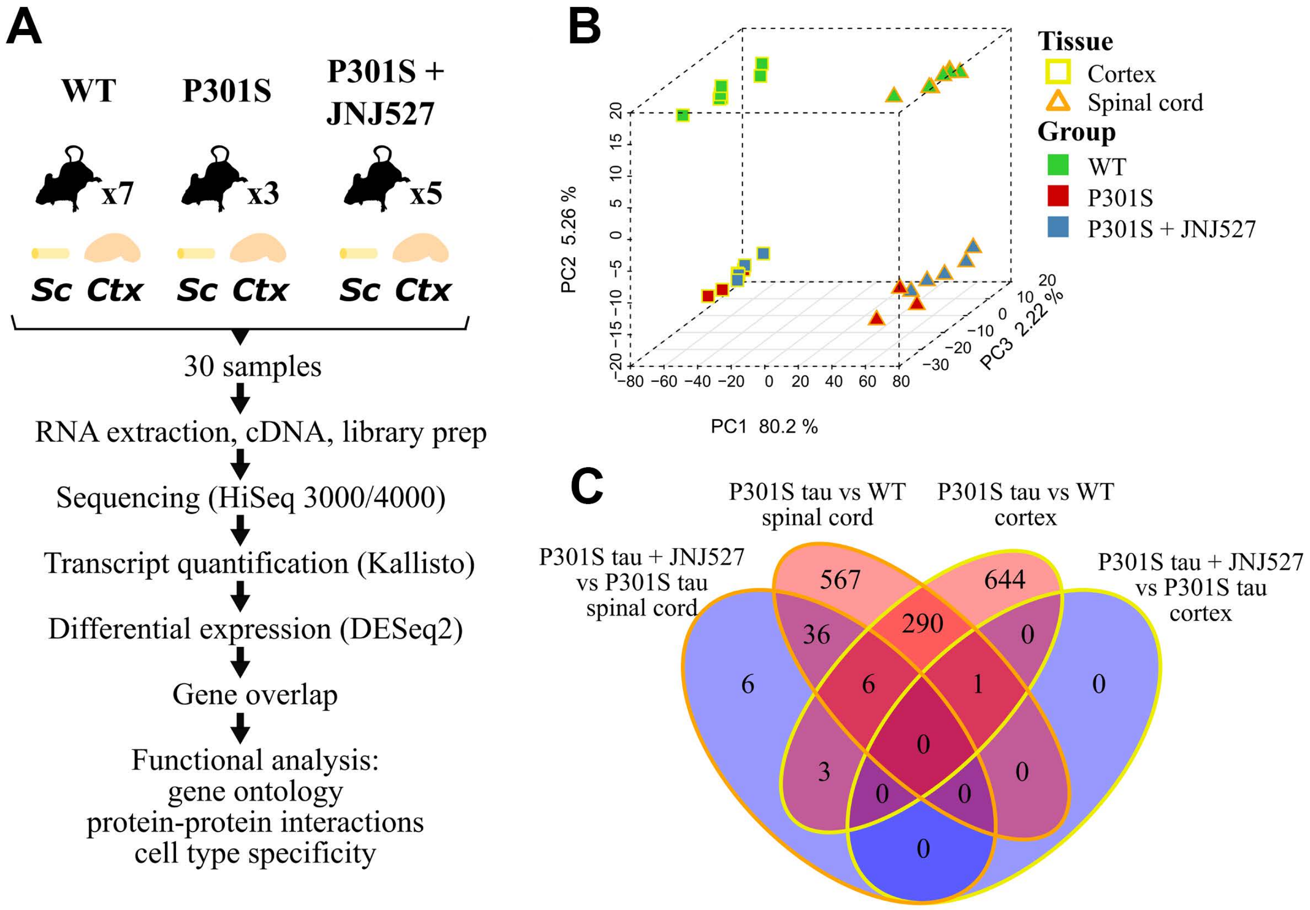

Figure S7. Experimental design for the transcriptomic analysis of P301S samples. (**A**) A total of 30 biological samples from both spinal cord (Sc) and cortex (Ctx) from 15 female mice were sequenced (wild type n=7, P301S n=3, P301S+JNJ-527 n=5). (**B**) First three principal components explaining together ~88% of the variance in the gene expression data. Note that the first principal component (PC1) separates the samples by tissue and second (PC2) by genotype (WT and P301S). (**C**) Differentially expressed genes between P301S and WT groups (disease signal/red), and between P301S+JNJ-527 and P301S groups (treatment signal/blue) were obtained separately for each tissue. The Venn diagram shows the overlap between differentially expressed genes in each comparison (FDR < 0.05).

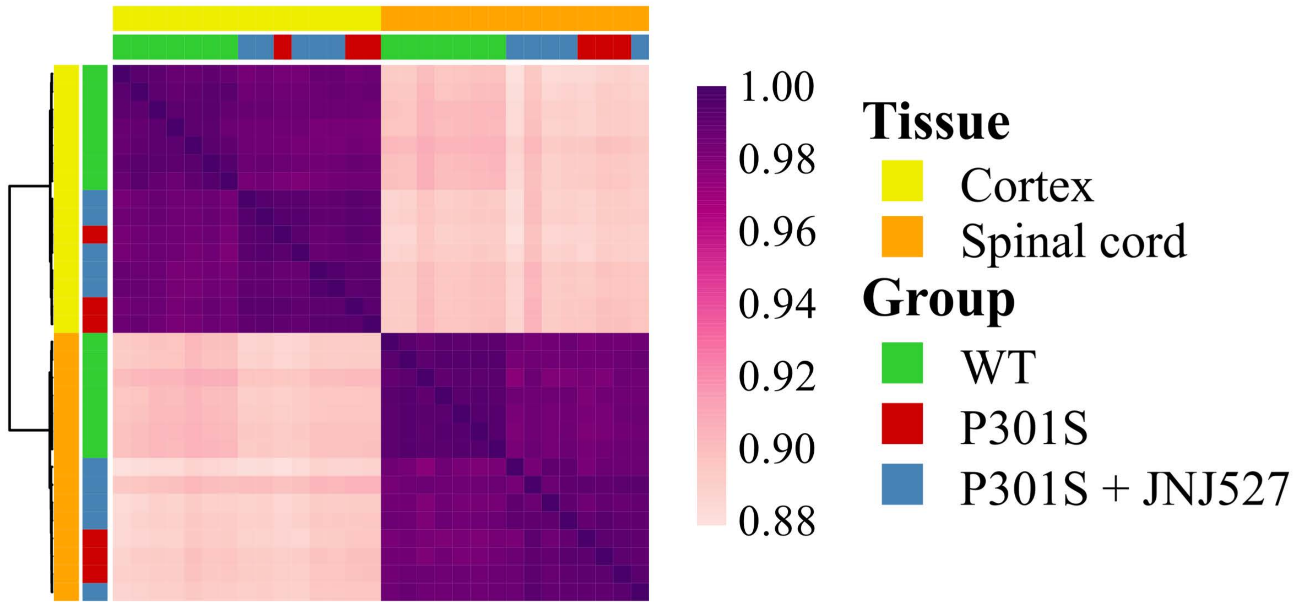

Figure S8. Gene expression similarity across 30 sequenced samples. Similarity is given as the Spearman correlation coefficients (rho) between each pair of samples. Across tissues, the correlation ranges from 0.878 to 0.907, and within tissue from 0.981 to 0.995, and 0.977 to 0.994 for cortex and spinal cord, respectively. Ward hierarchical clustering based on the Euclidean distance of 1-rho is represented by the tree.

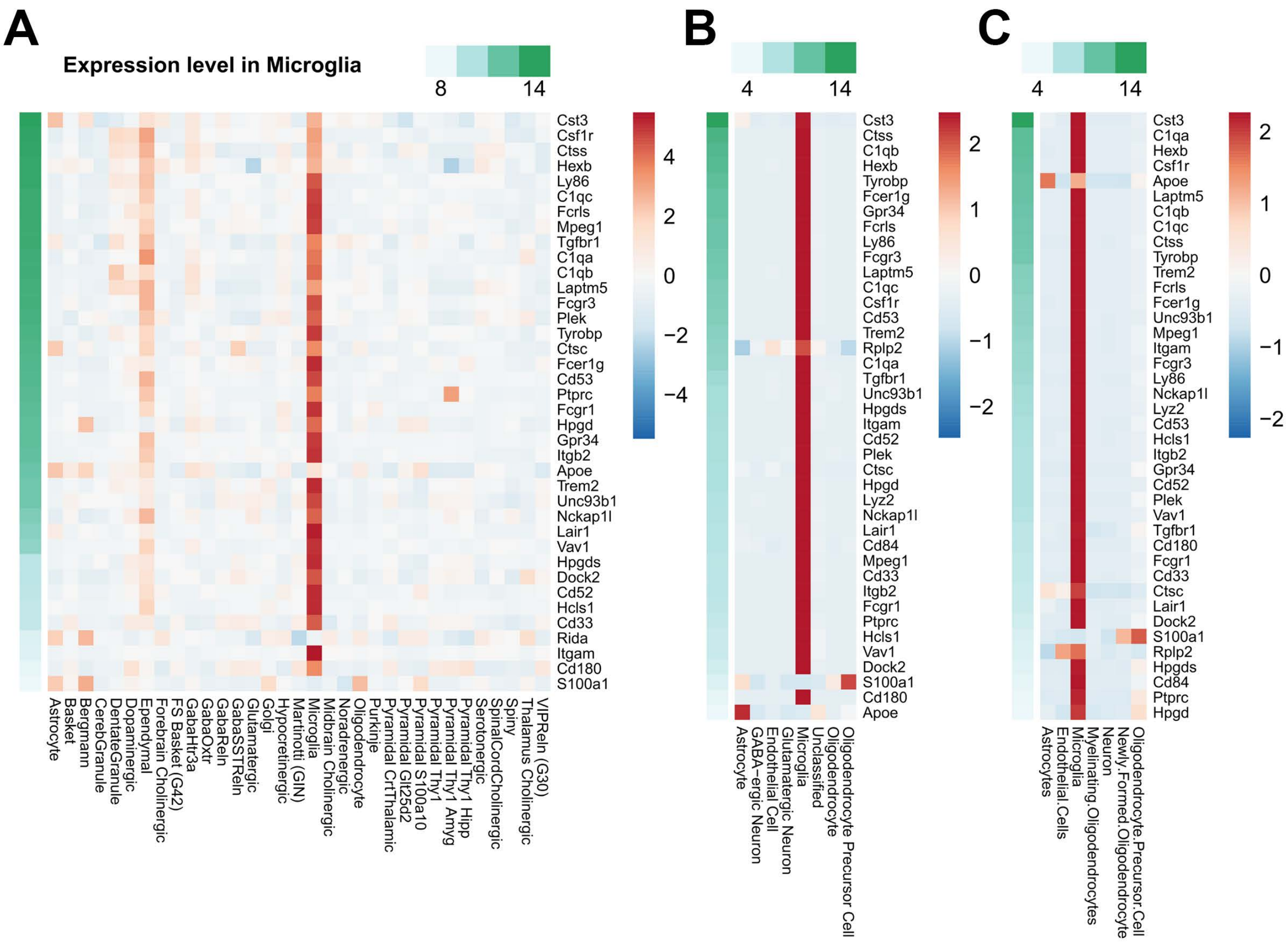

Figure S9. Microglial specific gene expression patterns among genes modulated by JNJ-527 in the spinal cord of P301S mice. (A) Gene expression patterns across diverse cell-types for 38 of the 41 genes that mapped the NeuroExpresso microarray dataset (Rplp2, Cd84, Lyz2 were not present in this dataset). Gene expression was averaged across samples of the same cell type and was then scaled per gene. Genes were sorted by their average expression level in microglial samples (shown in green).. (**B, C**) Confirmation of the microglial specific gene expression in two alternative datasets. RNA-seq gene expression patterns for multiple cell types from the Tasic dataset (Zerbino *et al.*, 2018) available through and from the RNA-Sequencing Transcriptome and Splicing Database of Glia, Neurons, and Vascular Cells of the Cerebral Cortex (Zhang *et al.*, 2014). Samples for the same cell type have been average and then scaled across cell-types. Genes are order by the gene expression in microglial samples in each dataset.
