# Supplementary figures and images for: CSF1R inhibitor JNJ-40346527 attenuates microglial proliferation and neurodegeneration in P301S mice
Source: Brain. 2019 Aug 26;142(10):3243–64. doi: 10.1093/brain/awz241 (PMC6794948; doi:10.1093/brain/awz241)

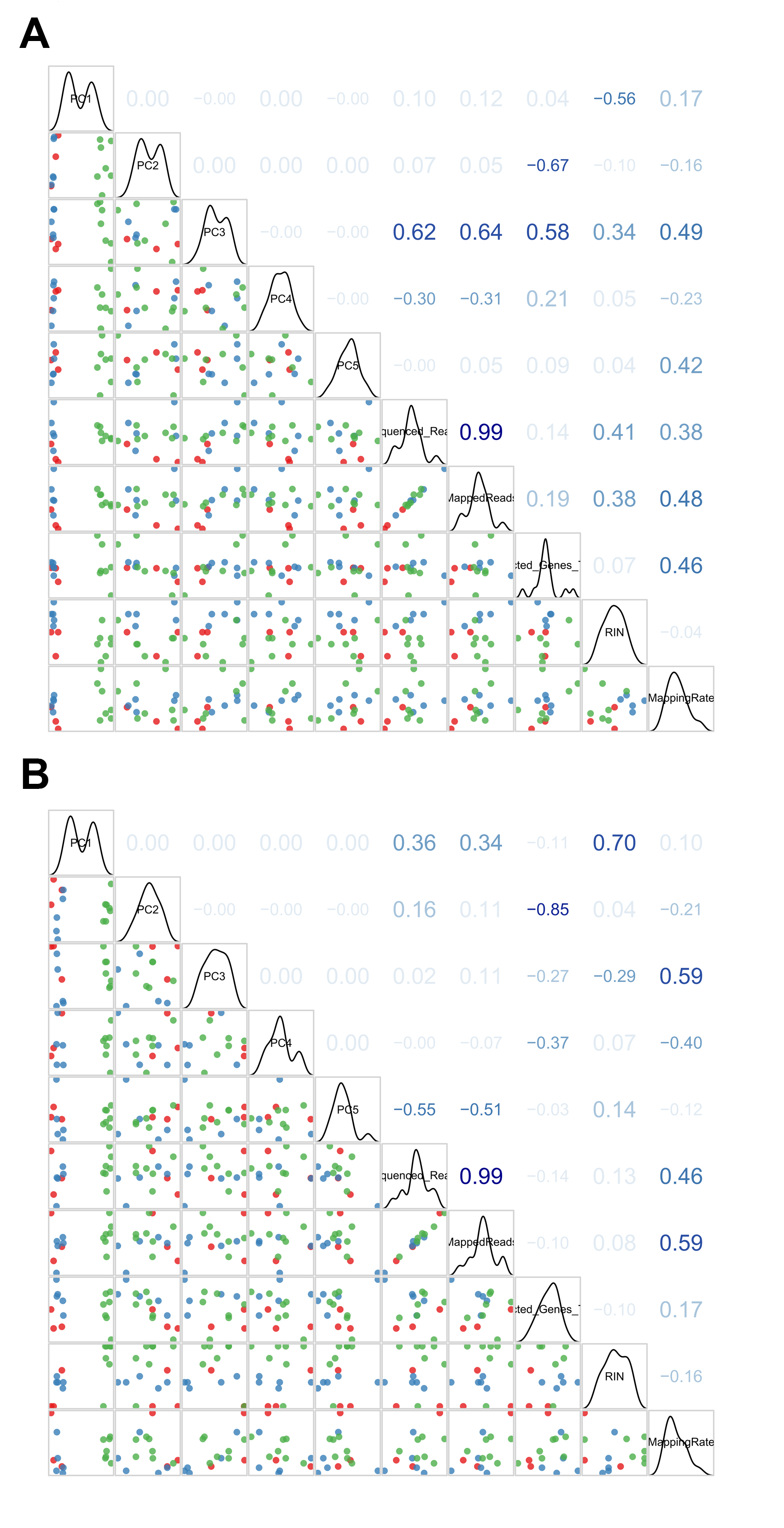

Supplement: awz241_Supplementary_Data [file awz241_supplementary_data.zip › awz241-Suppl_data/Supplementary_Figure S1.tif]

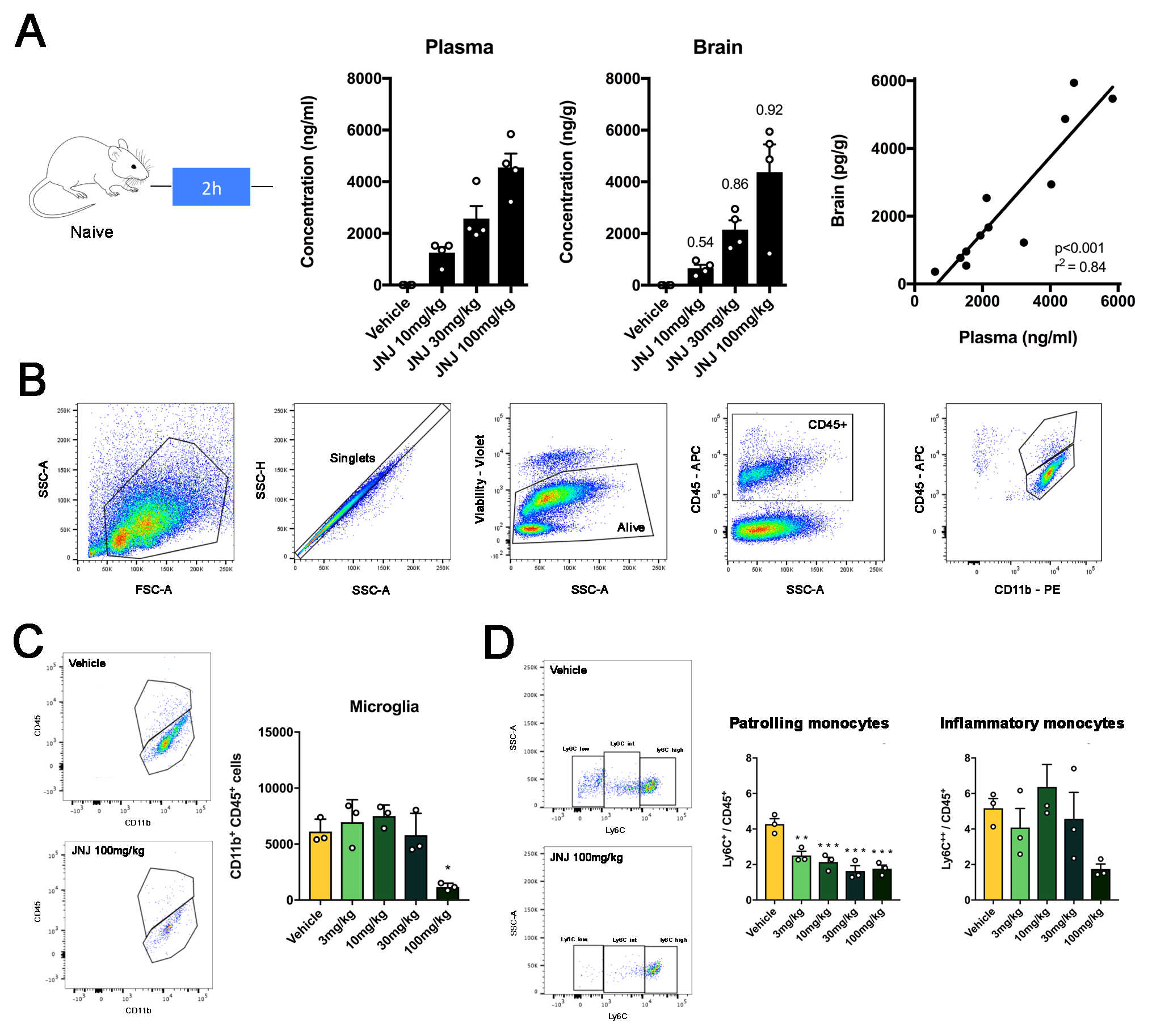

Supplement: awz241_Supplementary_Data [file awz241_supplementary_data.zip › awz241-Suppl_data/Supplementary_Figure S2.tif]

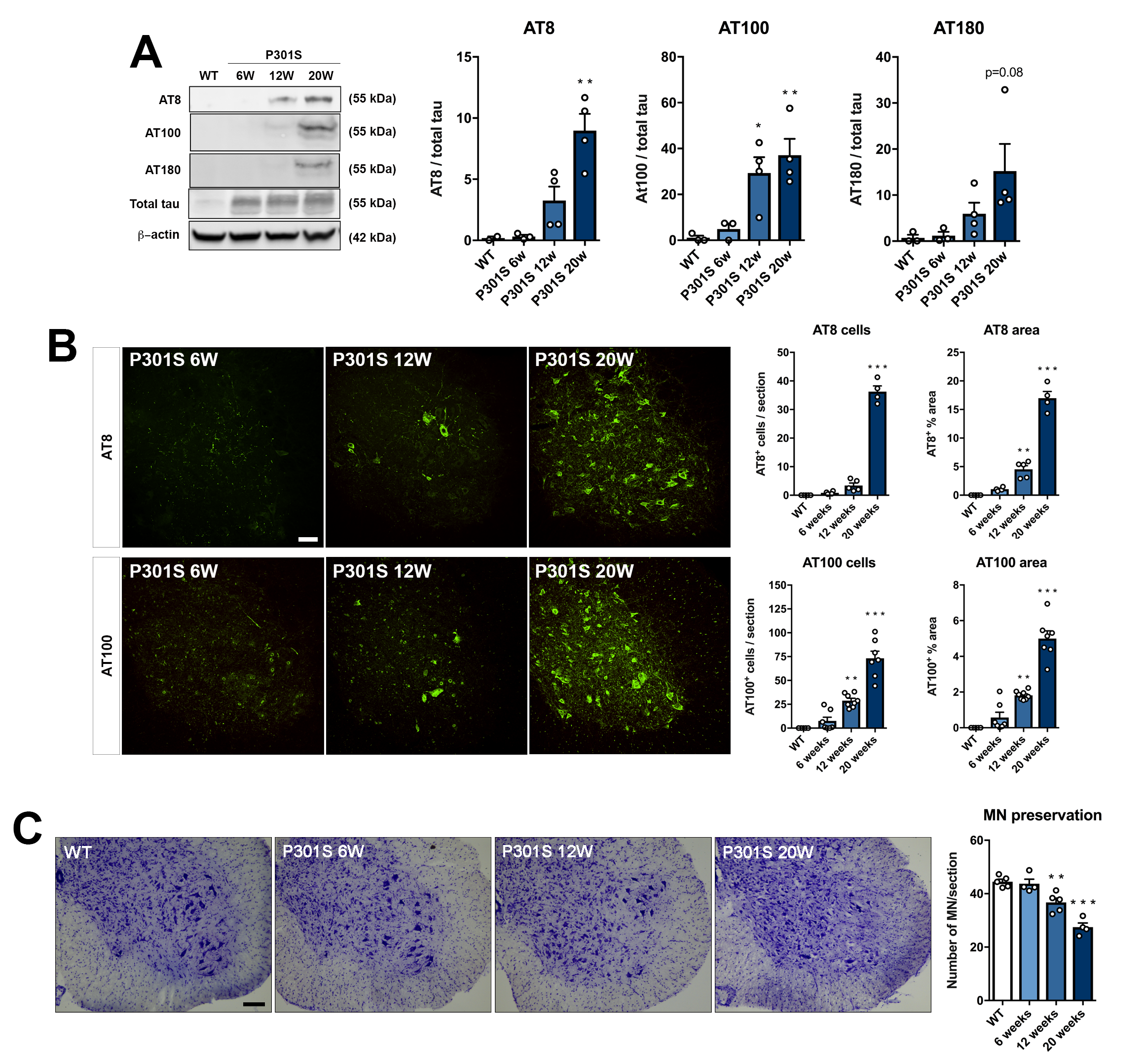

Supplement: awz241_Supplementary_Data [file awz241_supplementary_data.zip › awz241-Suppl_data/Supplementary_Figure S4.tif]

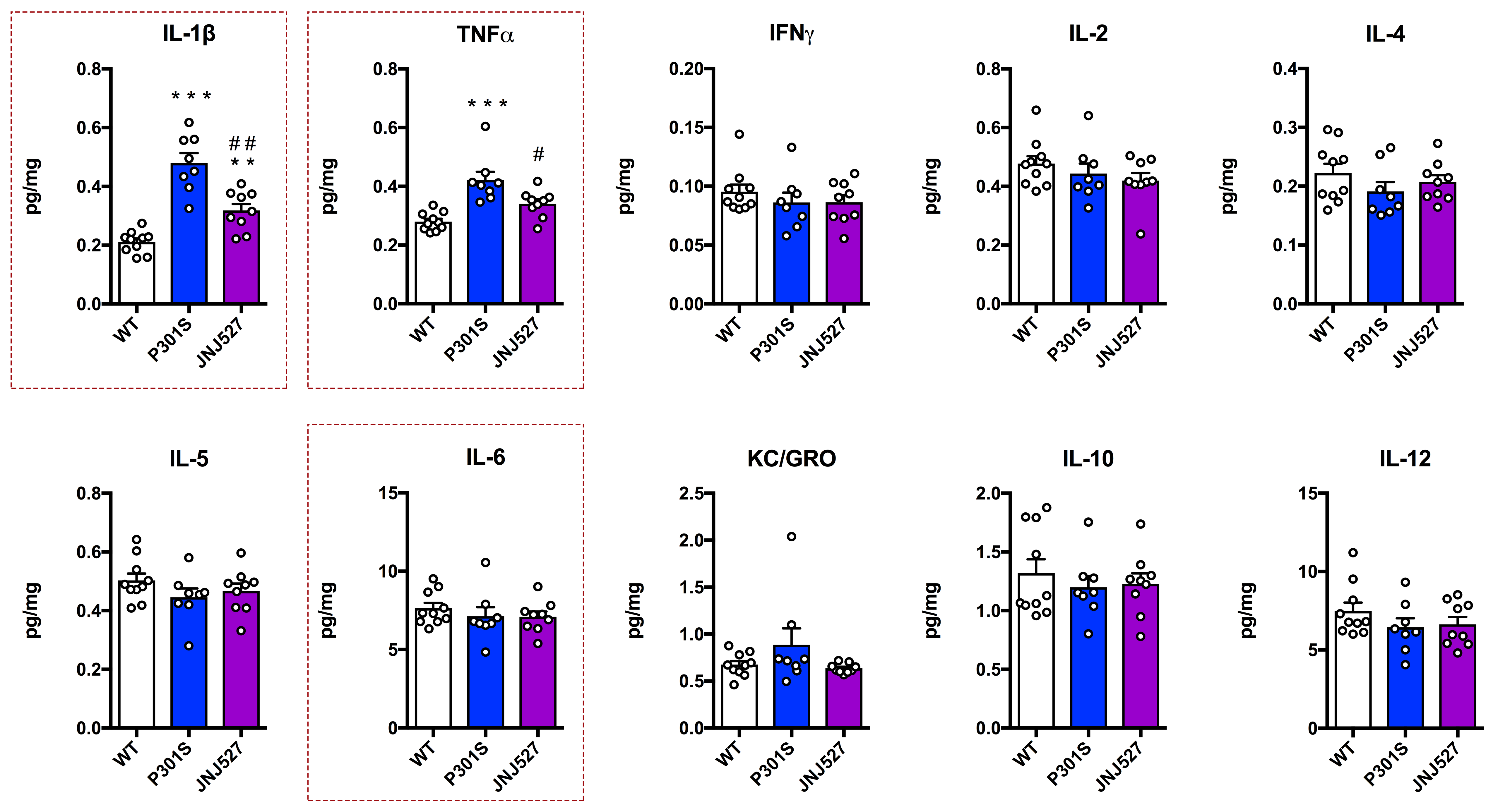

Supplement: awz241_Supplementary_Data [file awz241_supplementary_data.zip › awz241-Suppl_data/Supplementary_Figure S5.tif]

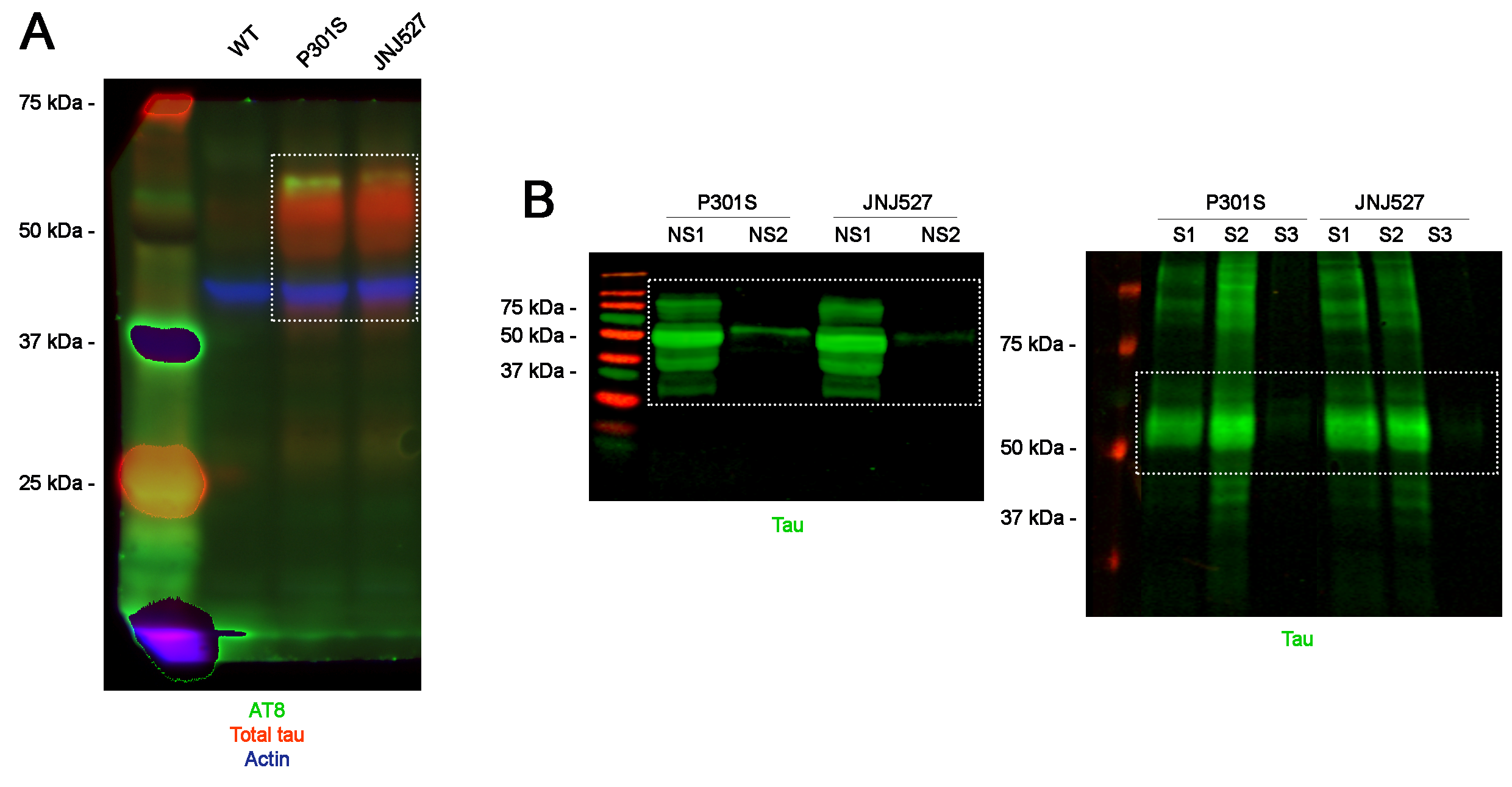

Supplement: awz241_Supplementary_Data [file awz241_supplementary_data.zip › awz241-Suppl_data/Supplementary_Figure S6.tif]

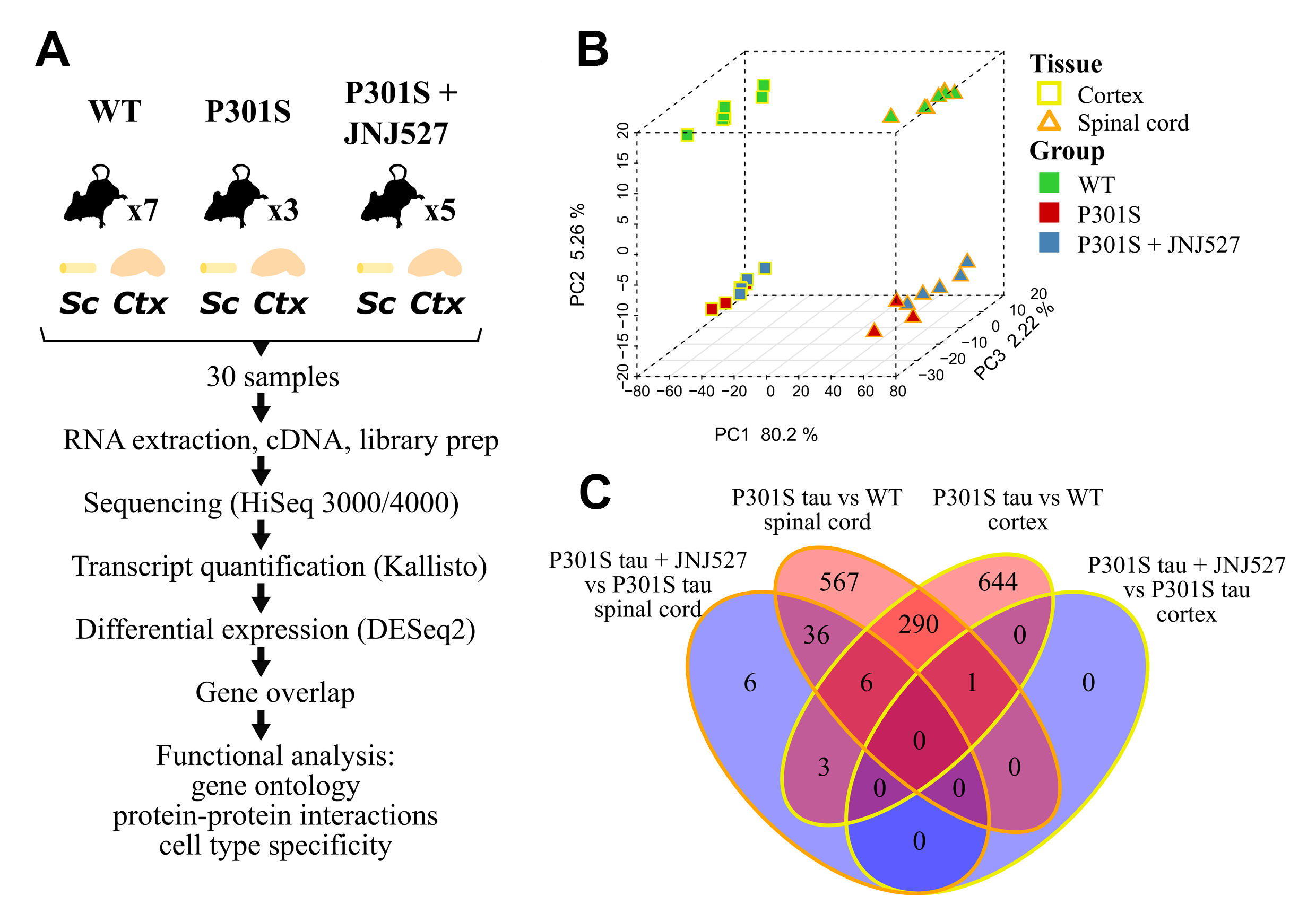

Supplement: awz241_Supplementary_Data [file awz241_supplementary_data.zip › awz241-Suppl_data/Supplementary_Figure S7.tif]

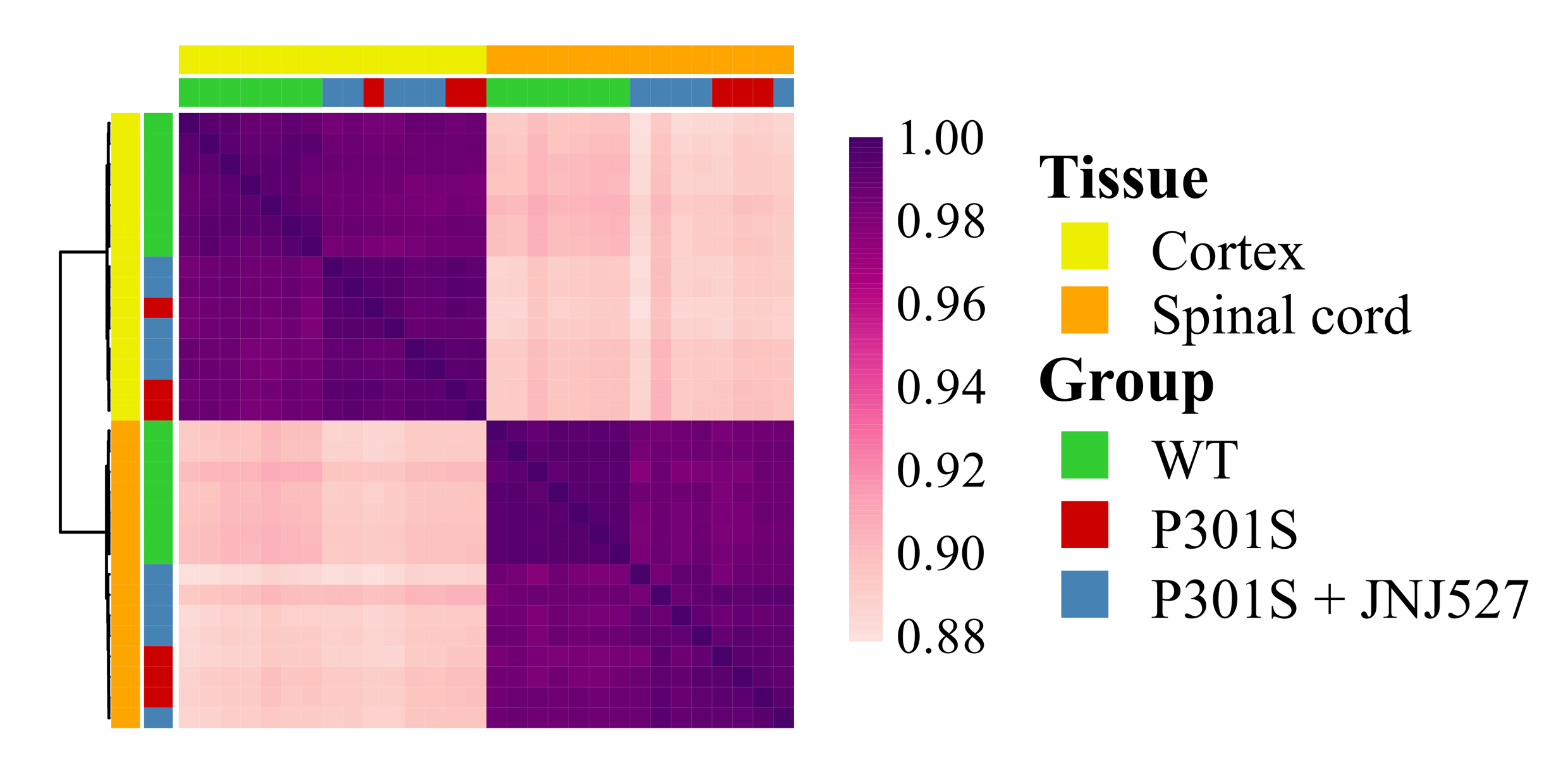

Supplement: awz241_Supplementary_Data [file awz241_supplementary_data.zip › awz241-Suppl_data/Supplementary_Figure S8.tif]

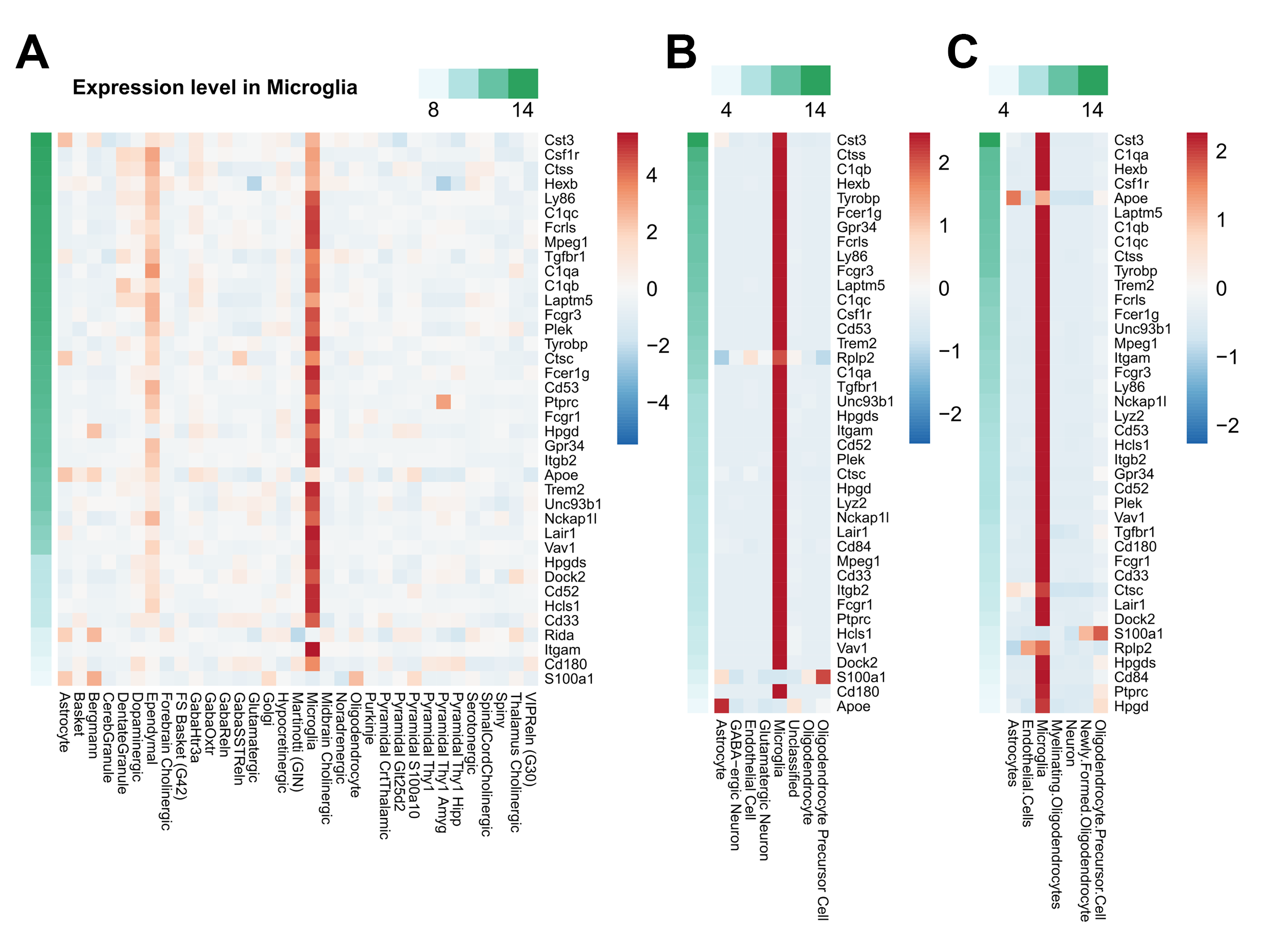

Supplement: awz241_Supplementary_Data [file awz241_supplementary_data.zip › awz241-Suppl_data/Supplementary_Figure S9.tif]
